# CD9‐association with PIP_2_ areas is regulated by a CD9 salt bridge

**DOI:** 10.1002/2211-5463.70084

**Published:** 2025-07-18

**Authors:** Yahya Homsi, Sara C. Konopka, Thorsten Lang

**Affiliations:** ^1^ University of Bonn, Faculty of Mathematics and Natural Sciences, Membrane Biochemiistry, Life & Medical Sciences (LIMES) Institute Germany

**Keywords:** EWI‐2, second messengers, signaling, tetraspanin‐enriched microdomains, tetraspanins

## Abstract

Tetraspanins are membrane proteins involved in multiple cellular functions, which they regulate by means of tetraspanin‐enriched microdomains. While many tetraspanin‐associated processes are regulated by intracellular signaling, possibly through the crosstalk between intracellular tetraspanin segments and second messengers like PIP_2_, this molecular crosstalk has remained largely unknown. To improve our understanding of this crosstalk, we investigate the possible relationship between an intracellular salt bridge of the tetraspanin CD9 and PIP_2_. We find that CD9 readily associates with PIP_2_‐rich areas, in contrast to its interaction partner EWI‐2. The opening of the CD9 salt bridge lowers the abundance of CD9 in these PIP_2_ areas. Instead, open‐CD9 can be located in regions that are more strongly populated with EWI‐2, promoting CD9‐EWI‐2 association. This study uncovers the process of an intracellular salt bridge regulating the association of CD9 with PIP_2_‐enriched areas. This points toward a possible link between intracellular tetraspanin segments and signaling.

## Graphical abstract

The tetraspanin CD9 has an intracellular salt bridge. If CD9 opens, open‐CD9 moves from PIP_2_‐rich areas to regions populated by its interaction partner EWI‐2. Hence, the state of the salt bridge regulates the distribution of CD9 and by this CD9‐EWI‐2 complex formation. It is tempting to speculate that PIP_2_ signaling may interfere with this equilibrium and by this regulates tetraspanins.

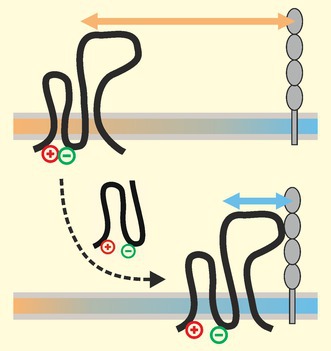

**Abbreviations**
PCC: Pearson correlation coefficient
PH: pleckstrin homology domain
PIP_2_
: Phosphatidylinositol bisphosphate
ROI: region of interest
SIL: small intracellular loop
STED: stimulated emission depletion
TEMs: tetraspanin‐enriched microdomains

Tetraspanins are a family of small membrane proteins forming primary complexes with a large variety of partner proteins, including immunoglobulin superfamily proteins, integrins, and proteases. Additionally, tetraspanins interact with one another [1]. This creates an extended interaction network referred to as the tetraspanin network [2], or tetraspanin web, which also contains tetraspanin‐enriched microdomains (TEMs). Due to their role in membrane micropatterning, tetraspanins are also known as ‘master organizers of the plasma membrane’ [3].

The diversity of tetraspanin binding partners implies that TEMs must be involved in a large variety of processes. Indeed, the list is long, including physiological and pathophysiological roles in (in alphabetical order) adhesion, cancer, cell–cell fusion, cell proliferation, extracellular vesicle formation, infectious diseases, migration, neurodegeneration, regulation of protease activity, signaling, spreading, and trafficking [4, 5, 6, 7, 8, 9, 10]. While it is generally agreed upon that tetraspanins organize functional TEMs, the exact nature of the aforementioned processes being regulated by TEMs still remains uncertain. Several ideas are discussed; for instance, TEMs may stabilize their interaction partners, assist in their folding, direct them to their cellular destination site, or build scaffolds for membrane curvature in trafficking [10, 11].

Many tetraspanin‐associated processes are regulated by intracellular signaling [12], which implies possible crosstalk between intracellular protein segments and second messengers like PIP_2_ or Ca^2+^. However, tetraspanins are constituted mainly of two extracellular loops and four transmembrane domains [13]. There is only one very short intracellular loop; consequently, it is aptly named as the small intracellular loop (SIL). A third of the SILs in the human tetraspanins is composed of only six amino acids [13], which is sufficient to form a U‐turn. Apart from a few exceptions, the intracellular N‐ and C‐terminal peptides can be identified as short as well [13]. Hence, in most tetraspanins, only short amino acid stretches are oriented toward the intracellular side. This suggests that direct interactions with second messengers would be rather mediated via an electrostatic bond than binding into a pocket formed of many amino acids.

Our knowledge about the possible roles of these intracellular segments is limited, with the exception that palmitoylated cysteines are crucial for the assembly of the tetraspanin web [14]. For instance, the SIL of the tetraspanin CD82 harbors one palmitoylation site. Its elimination strongly diminishes the association of CD82 with human T‐cell lymphotrophic virus type 1 Gag protein [15]. Interestingly, the same effect is observed when a glutamate (E) of the SIL is mutated [15]. Analysis of the SIL sequence in five species reveals that this glutamate is conserved in four animal tetraspanin families but not in *Arabidopsis* [16]. The glutamate is not the only conserved amino acid but part of a conserved five amino acid REXXC sequence. During translation, the first positively charged amino acid of the sequence, the arginine (R), likely aids in correct membrane insertion. The cysteine (C) of the fifth position is supposed to stabilize TEMs by being palmitoylated [16], which is in accordance with the observation that depalmitoylation weakens the above‐mentioned CD82 interaction with human T‐cell lymphotrophic virus type 1 Gag protein [15]. However, how the glutamate functions remains unclear.

In the CD9 crystal structure, the respective negatively charged glutamate (E84) of the CD9‐SIL forms a salt bridge with a positively charged lysine (K11) of the N‐terminal peptide [16]. The salt bridge maybe an artifact of protein crystallization. Yet, the association between CD9 and its primary interaction partner EWI‐2 is increased upon elimination of the salt bridge, by either exchanging the glutamate (CD9‐E84A) or the N‐terminal lysine to alanine (CD9‐K11A). Interchanging of the salt bridge charges (CD9‐K11E/E84K) has no effect on the interaction, reinforcing the idea that the observed effects indeed rely on a salt bridge [16]. It remains to be investigated how the salt bridge mechanistically promotes CD9‐EWI‐2 association. An explanation could be an increased CD9‐EWI‐2 binding affinity in the open salt bridge conformation. Alternatively, the lateral CD9 distribution may be regulated by the salt bridge, enabling more productive encounters between open‐CD9 and EWI‐2, which in theory should elevate CD9‐EWI‐2 complexes also without an increased binding affinity.

At present, it is unclear whether molecular crosstalk between intracellular tetraspanin segments and second messengers as PIP_2_ does exist. Regarding the CD9/CD81 interaction partner EWI‐2 [17], it has been shown that the short EWI‐2 cytoplasmic tail contains a cluster of five positively charged amino acids interacting with phosphoinositides, including PIP_2_ [18]. For other proteins, it has been suggested that the second messenger PIP_2_ binds electrostatically to N‐terminal positively charged lysines (e.g., see [19]). The N‐terminus of CD9 contains three lysines (K4, K8, and K11, the latter forming the salt bridge) which, according to the electrostatic binding model, could possibly interact with PIP_2_.

To shed some light on the relationship between CD9 and the second messenger PI(4,5)P_2_, we have studied whether CD9 and its interaction partner EWI‐2 [17] are associated with PI(4,5)P_2_ areas, and whether association is dependent on the salt bridge. We find that CD9 associates more strongly with PI(4,5)P_2_‐rich areas than EWI‐2, and that the opening of the salt bridge transfers CD9 from PI(4,5)P_2_‐rich areas to regions where EWI‐2 is more abundant. This suggests that the salt bridge regulates the lateral distribution of CD9 and consequently the formation of CD9‐EWI‐2 complexes.

## Materials and methods

### Antibodies and plasmids

Plasmids for protein expression encoding the following fusion proteins were used: mCherry fused to the N‐terminus of the human PH domain of phospholipase Cδ (NM_006225.4) [20]. A monomeric variant of GFP fused to the C‐terminus of human CD9 [16], CD9‐K11A [16], CD9‐E84A [16], CD9‐K11E/E84K [16], and EWI‐2 [21]. A monomeric variant of RFP fused to the C‐terminus of human EWI‐2 [22], CD9 [22], CD81 [22], and CD151‐RFP [23]. The primary antibody used for co‐patching was a rabbit polyclonal anti‐GFP antibody (ab290; Abcam, Cambridge, UK), and as a secondary antibody, we used donkey‐anti‐rabbit coupled to Alexa Fluor 647 (A31573; Invitrogen, Carlsbad, CA, USA). As control antibodies, we used rabbit polyclonal anti‐EEA1 (early endosome antigen 1; sc‐33 585, Santa Cruz, Dallas, TX, USA) and goat polyclonal anti‐EGFR (epidermal growth factor receptor; sc‐03‐G, Santa Cruz) in combination with the above‐mentioned secondary antibody or rabbit‐anti‐goat coupled with CF647 (20 049; Biotium, Fremont, CA, USA). For western blot analysis, the primary antibodies used were a mouse monoclonal anti‐CD9 antibody (clone MM2/57, CBL162; Merck Millipore, Darmstadt, Germany) and a rabbit polyclonal anti‐GFP antibody (pabg1; Chromotek, Planegg‐Martinsried, Germany). Secondary antibodies used for western blot analysis were IRDye 800CW goat anti‐mouse (926–32 210) and IRDye 680RD goat anti‐rabbit (926–68 071) both from (LI‐COR Biotechnology, Bad Homburg, Germany).

### Cell culture and membrane sheets

HaCaT cells [24] were cultured in high glucose DMEM (Gibco 61 965–026, Thermo Fisher Scientific, Waltham, MA, USA) supplemented with 10% FBS (PAN Biotech, Aidenach, Germany, P30‐3031) and 1% Penicillin/Streptomycin [10 000 U·mL^−1^ Penicillin, 10 mg·mL^−1^ Streptomycin (PAN Biotech, P06‐07100)] at 37 °C with 5% CO_2_ and passaged after trypsination. For transfection, 2 × 10^6^ cells were mixed with 15 μg plasmid per construct (with the exception of Fig. S4 for which 7.5 μg per construct were used) in cytomix solution (120 mm KCl, 10 mm KH_2_PO_4_, 10 mm K_2_HPO_4_, 0.15 mm CaCl_2_, 2 mm EGTA, 5 mm MgCl_2_, and 25 mm HEPES‐KOH, pH 7.6) for electroporation (250 V, 1500 μF, and infinite Ω) in a 2 mm cuvette using a Gene pulser Xcell electroporation system (Bio‐Rad, Hercules, CA, USA). Afterward, cells were seeded onto poly‐L‐lysine coated glass‐coverslips as previously described [22]. One day after transfection, membrane sheets were generated in ice‐cold sonication buffer (120 mm potassium glutamate, 20 mm potassium acetate, 10 mm EGTA, 20 mm HEPES, pH 7.2). To this end, a distance to the sonication tip of about 5 mm was adjusted. Then, moving around the coverslip, we applied at four different locations each a 100 ms ultrasound pulse at 80% power (employing a Bandelin Sonoplus GM2070 sonifier). The membranes were either directly fixed with 4% PFA in PBS (137 mm NaCl, 2.7 mm KCl, 1.76 mm KH_2_PO_4_, and 10 mm Na_2_HPO_4_, pH7.4) for 45 min, or after a 5 min incubation at room temperature in 1% BSA‐PBS [PBS containing 1% bovine serum albumin (BSA)] without or with anti‐GFP antibody or control antibodies (diluted 1 : 50). Subsequently, PFA was quenched with 50 mm NH_4_Cl in PBS for 30 min and samples were incubated with corresponding secondary antibodies (diluted 1 : 500) in 1% BSA‐PBS for 15 min, followed by three washes. When applying 300 mm sucrose, prior to membrane sheet generation, medium was exchanged by prewarmed medium supplemented with 300 mm sucrose, followed by a 15 min incubation at 37 °C with 5% CO_2_.

### Epi‐fluorescence microscopy

Epi‐fluorescence microscopy was performed as previously described [22], with the exception that a different illumination system was used (SPECTRA X Light Engine; Lumencor, Beaverton, OR, USA). Membrane sheets were imaged in PBS containing TMA‐DPH [1‐(4‐trimethylammoniumphenyl)‐6‐phenyl‐1,3,5‐hexatriene p‐toluenesulfonate] (T204; Thermo Fisher Scientific) for visualizing the membranes in the blue channel. GFP, RFP/mCherry, and Alexa Fluor 647/CF647 were recorded in the green, red, and long‐red channel, respectively.

**Fig. 1:**
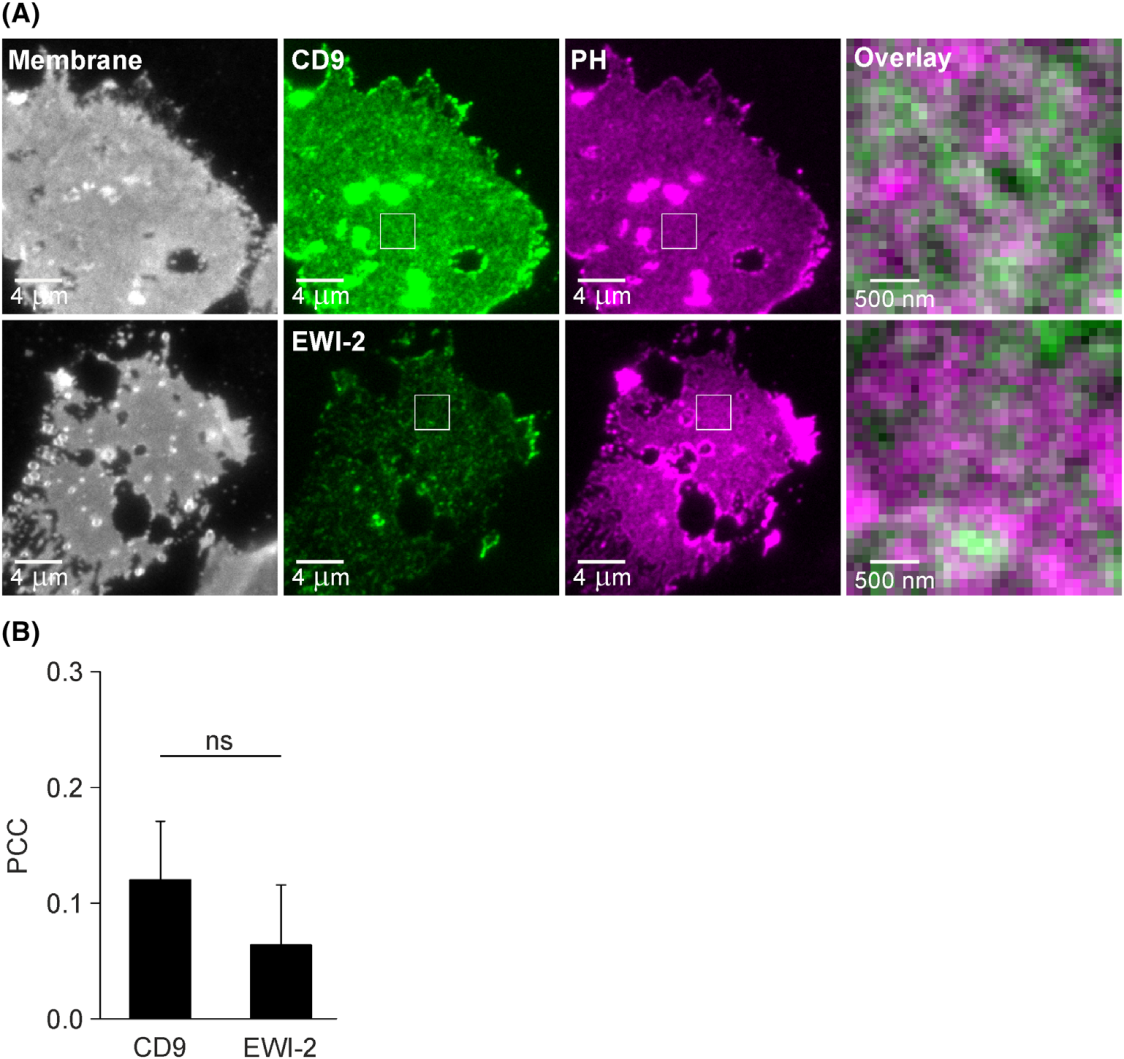
Pearson correlation coefficient (PCC) between PH and CD9 or EWI‐2 analyzed on directly fixed basal plasma membrane sheets. (A) HaCaT cells were double transfected with PH (the pleckstrin homology domain of phospholipase Cδ1 fused to mCherry) and CD9 or EWI‐2 (each fused to monomeric GFP). One day after transfection, membrane sheets were generated, fixed, and imaged. Left column, membrane visualization by the dye TMA‐DPH (displayed using a grayscale lookup table); middle columns, CD9 or EWI‐2 (green lookup table) and PH (magenta lookup table); right, magnified views of overlays from the boxed regions of the middle images. With the exception of the TMA‐DPH images (left), images of one channel are shown at the same settings of brightness and contrast. (B) The average PCC between PH and CD9 or EWI‐2 analyzed in squared regions of interest (ROIs) placed onto central membrane areas devoid of holes or large fluorescent accumulations. Values are given as means ± SD (*n* = 6 biological replicates; each replicate and condition includes 3–13 analyzed membrane sheets; four out of the six replicates are analyzed separately in Figs 4 and 5). Statistical test: unpaired two‐tailed Student's *t*‐test (ns: non‐significant).

For image analysis, we used Fiji ImageJ [25]. In the blue channel (TMA‐DPH) visualizing the membranes, we identified a membrane region devoid of holes, where a squared region of interest (ROI) was placed. Then, the ROI was transferred to the green and red channels for measuring the average intensity. From the average intensities, the background values were subtracted using ROIs placed next to the membranes. The PCC was calculated using the ImageJ plugin Colocalization_Indices. For the validation of small differences in the average PCCs in Figs 1, 7 and 8, each PCC value was validated using the ImageJ plugin Coloc2 employing the Costes test (scrambling blocks of pixels [26] in the mCherry channel) using 4 pixels (1 pixel = 83.3 nm) as PSF size. In case the Costes *P*‐value was < 0.95, we excluded the PCC from the analysis.

### STED microscopy

For STED microscopy, we used samples that, after imaging them by epi‐fluorescence microscopy, were incubated for 1 h at room temperature with nanobodies (GFP‐booster coupled to Atto647N (gba647n‐100) and RFP booster coupled to Atto594 (rba594‐100) both from Chromotek) diluted 1 : 100 in 3% BSA‐PBS. Subsequently, membranes were washed and embedded in ProLong Gold antifade mountant (P36930; Invitrogen). For imaging, we employed a 4‐channel STED microscope from Abberior Instruments (Göttingen, Germany). The microscope is based on an Olympus IX83 confocal microscope equipped with a UPlanSApo 100× (1.4 NA) objective (Olympus, Tokyo, Japan). We used a 561 nm [at 50%; detected at 580–630 nm (red channel); 3 line steps] and a 640 nm [at 50%; detected at 650–720 nm (long‐red channel); 3 line steps] laser for excitation, and a 775 nm laser (at 25%) for depletion. The pixel size was 25 nm.

For image analysis, we used Fiji ImageJ (see above) in combination with a macro previously described [21]. In brief, after correction of the long‐red channel for 50% crosstalk from the red channel, a Gaussian blur (*σ* = 0.5) was applied for noise reduction. Then, local maxima were detected in squared ROIs using the function ‘Find maxima’ (noise tolerance 2 for CD9‐RFP and 4 for CD9‐GFP/CD9‐E84A‐GFP) that yields maxima positions as coordinates (pixel positions). Onto each maximum position, a circular ROI with a diameter of 5 pixel was placed, that determined in the ROI the ‘center of mass’, yielding for each maximum a sub‐pixel position. In addition, onto each maximum position, either a horizontal or a vertical linescan (31 pixels long × 3 pixels width) was placed. A Gaussian function was fitted to the intensity distribution measured by the linescans. Maxima in the CD9‐RFP channel were selected if, at least for one linescan, they had a fit quality of *R* ^2^ > 0.7 and a peak in the middle third of the linescan. Using the sub‐pixel positions of the selected CD9‐RFP maxima, we determined in the CD9‐GFP/CD9‐E84A‐GFP channel the distance to the next nearest maxima at sub‐pixel position. For the generation of a control distribution with purely random distances, we flipped the image of CD9‐GFP/CD9‐E84A‐GFP. Using the same selected CD9‐RFP positions as above, we determined in the flipped images the next nearest maxima at sub‐pixel position. The same analysis was performed with three more noise tolerance settings: 4 for CD9‐RFP and 8 for CD9‐GFP/CD9‐E84A‐GFP, 6 for CD9‐RFP and 12 for CD9‐GFP/CD9‐E84A‐GFP, 2 for CD9‐RFP and 12 for CD9‐GFP/CD9‐E84A‐GFP.

The PCC between CD9‐GFP and its nanobody labelling was determined from squared ROIs using a custom‐written ImageJ macro.

### Immunoprecipitation

HaCaT cells were transfected with CD9‐GFP or CD9‐E84A‐GFP and plated in 10 cm dishes. As control, we plated the same amount of untransfected cells. After 1 day, cells were washed once with 5 mL ice‐cold TRIS buffer (10 mm TRIS, 150 mm NaCl, 5 mm CaCl_2_, and 5 mm MgCl_2_, pH 7.4) and scraped from the dishes in 1 mL TRIS‐lysis buffer [TRIS buffer supplemented with 1% Brij 97 (P6136; Sigma‐Aldrich, Taufkirchen, Germany), protease inhibitor cocktail (11697498001, Roche, Mannheim, Germany), and 1 mm PMSF] using a cell scraper. Then, the suspension was transferred into a 1.5‐mL tube and lysed further for 30 min at 4 °C under rotation. Afterward, cell lysates were centrifuged at 4 °C for 5 min at 3800 ***g***. The supernatants were used for immunoprecipitation, adding 25 μL GFP‐Trap Agarose beads (gta‐100; Chromotek, Planegg‐Martinsried, Germany) to 1 mL lysate, and the suspension was rotated at 4 °C for 1 h. Afterward, beads were collected by centrifugation for 2 min at 2500 ***g*** and washed twice with TRIS buffer supplemented with protease inhibitors and 1 mm PMSF. The washed beads were then processed for western blot analysis under non‐reducing conditions. To this end, beads were boiled at 95 °C for 5 min in 2 × Laemmli sample buffer without β‐mercaptoethanol, before loading them onto a 10% SDS‐PAGE gel. After transferring proteins from the gel to a nitrocellulose membrane, the membrane was blocked for 1 h at room temperature with Intercept blocking buffer (927–60 001; LI‐COR Biotechnology, Bad Homburg, Germany). Then, in a first round of immunolabeling, the membrane was incubated with CD9 primary antibody (1 : 1000 in Intercept blocking buffer) overnight at 4 °C, followed by the secondary antibody IRDye 800CW goat anti‐mouse (1 : 5000 in Intercept blocking buffer) for 1 h at room temperature, followed by imaging using a ChemiDoc MP Imaging System (Bio‐Rad Laboratories, Inc., Hercules, California, USA). Then, in a second round of immunolabeling, the membrane was incubated with anti‐GFP antibody (1 : 5000 in Intercept blocking buffer) followed by IRDye 680RD goat anti‐rabbit antibody (1 : 5000 in Intercept blocking buffer) each for 1 h at room temperature, followed by imaging as above. Images were recorded and saved as 16‐bit TIFFs. Signal quantification was performed using ImageJ by measuring integrated intensities of the bands of immunoprecipitated GFP and co‐precipitated endogenous CD9 (CD9_endo_). Co‐precipitated CD9_endo_ band integrated densities were related to the respective GFP integrated band intensities. The CD9_endo_/CD9‐E84A‐GFP ratios were normalized to the CD9_endo_/CD9‐GFP ratios (set to 100%).

### Statistics

Significances were calculated with the program graphpad Prism 9.5.1 (graphpad Software; LLC) using the one‐way ANOVA test followed by Dunnett's multiple comparison test, or the two‐way ANOVA test followed by Šidák's multiple comparisons test. Two groups were compared by the unpaired two‐tailed Student's *t*‐test (Microsoft Excel; microsoft Corporation) or by calculation of the confidence interval (graphpad Prism 9.5.1; graphpad Software, LL), if data were normalized to control. ns, non‐significant; *P < 0.05; **P < 0.01; ***P < 0.001; ****P < 0.0001. Linear regression lines were generated and analyzed using the software originpro 8g (OriginLab Corporation, Northampton, MA, USA).

## Results

### CD9 is associated with PIP_2_ ‐rich areas

In comparison with the size of a protein, the very small PI(4,5)P_2_ molecule must be labelled fluorescently in order to microscopically study the association of CD9 with PI(4,5)P_2_ areas. However, direct attachment of a fluorophore to PI(4,5)P_2_ would ultimately not visualize only PI(4,5)P_2_, as PI(4,5)P_2_ is converted rapidly to other phosphoinositides by kinases and phosphatases [27]. Moreover, covalent attachment of fluorophores to lipids may change their lateral dynamics. Finally, selective and efficient incorporation of labelled PI(4,5)P_2_ into the intracellular membrane leaflet would pose a challenge. For these reasons, the lateral distribution of phosphoinositide pools is studied by means of fluorescent protein‐labelled sensors recognizing different phosphoinositides as, for example, PIP, PIP_2_, or PIP_3_ [28]. For PI(4,5)P_2_, the human pleckstrin homology domain of phospholipase Cδ1 (PLCδ‐PH) is used, fused N‐terminally to a fluorescent protein [29], as, for example, a monomeric red fluorescent protein variant (mCherry‐PLCδ‐PH, [20]). As PH binds with high specificity to PI(4,5)P_2_, its fluorescent mCherry‐tag reports on PI(4,5)P_2_‐enriched domains. In the following, for simplicity's sake, we refer to mCherry‐PLCδ‐PH as PH and to PI(4,5)P_2_ as PIP_2_.

**Fig. 2:**
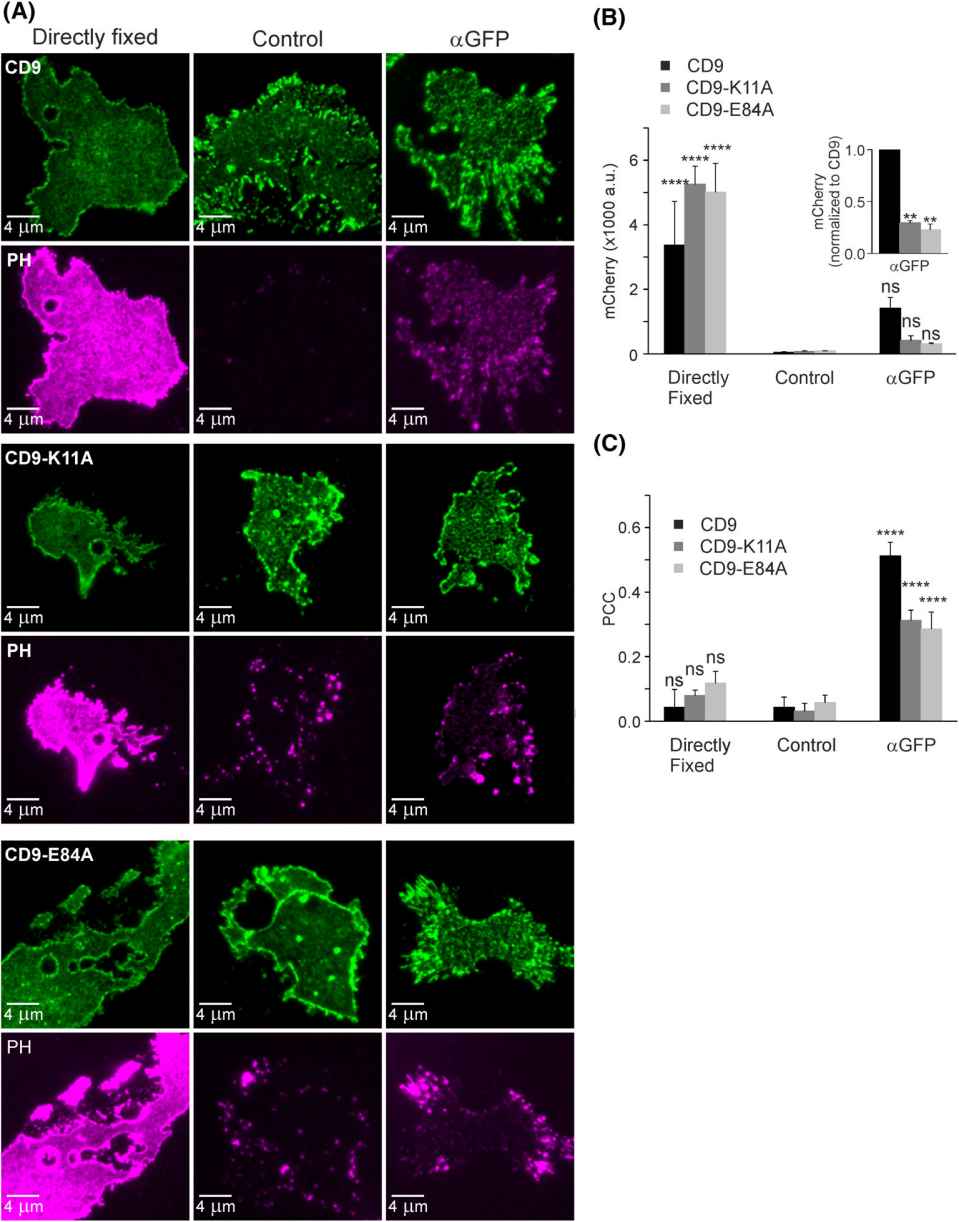
Antibody‐induced patching of CD9, CD9‐K11A and CD9‐E84A. (A) HaCaT cells were double transfected with PH and GFP‐labelled CD9 (top), CD9‐K11A (middle), or CD9‐E84A (bottom). One day after transfection, membrane sheets were generated and either directly fixed (left panels) or after a 5‐min incubation at RT with control buffer (Control, middle panels) or an anti‐GFP antibody (αGFP, right panels). A green lookup table is used for CD9, CD9‐K11A, and CD9‐E84A, and a magenta lookup table for PH. Images of one channel are shown at the same settings of brightness and contrast. In parallel, we performed control experiments: (i) employing the GFP antibody on membrane sheets of cells only expressing PH or (ii) employing different polyclonal antibodies (Fig. S3). (B) The average membrane‐associated PH (mCherry‐intensity) analyzed with squared regions of interest (ROIs). The inset shows the αGFP condition after normalization of CD9‐K11A and CD9‐E84A to CD9. (C) PCCs analyzed between PH and CD9, CD9‐K11A, and CD9‐E84A with squared ROIs. Values are given as means ± SD (*n* = 3 biological replicates; each replicate and condition includes 13–28 analyzed membrane sheets). Statistical test: two‐way ANOVA test followed by Šidák's multiple comparisons test comparing in (B) and (C) control to directly fixed or αGFP (ns: non‐significant; ****P < 0.0001). (B) Inset, the confidence interval was calculated for the comparison of CD9‐K11A to CD9 (**P < 0.01) or CD9‐E84A to CD9 (**P < 0.01).

PH binding to PIP_2_ is reversible, resulting in an equilibrium between cytosolic and membrane‐bound PH (see also Fig. S1). It should be noted that after PH binding, PIP_2_ is masked and can no longer be metabolized or interact with other proteins. However, this state is only transient (all PH molecules dissociate from the membrane within minutes; compare directly fixed and control in Figs 2B, 4B and 6A). Therefore, membrane‐bound PH is supposed to visualize the lateral distribution of PIP_2_ [29].

For examining the amount of PH at the plasma membrane and its lateral distribution, we employ unroofed cells [30], also referred to as plasma membrane sheets. The upper parts of the cells are removed by sonication pulses, leaving behind the basal plasma membrane (Fig. S1). To get an impression of the plasma membrane shape, we visualize membranes by the dye TMA‐DPH (Fig. 1A, left panels).

First, we examine the overlap between PIP_2_‐rich areas (as visualized by PH) and CD9, or its primary interaction partner EWI‐2. To this end, HaCaT cells are double transfected with PH and CD9 or EWI‐2 (fused to a monomeric variant of GFP). One day after transfection, membrane sheets are generated, paraformaldehyde fixed, and recorded by microscopy. In the recordings, PH is highly abundant, and not as uniformly distributed as the membrane marker TMA‐DPH (Fig. 1A, compare magenta and gray). CD9 and EWI‐2 form more punctate patterns than PH (Fig. 1A, compare green and magenta). For analyzing the similarity of the images, we calculate the Pearson correlation coefficient (PCC) between PH and CD9 or PH and EWI‐2. In principle, it can adopt a value of 1 if images overlap perfectly, 0 if images are unrelated, and −1 for an image and its negative. For CD9 and EWI‐2, we obtain low PCCs of 0.12 and 0.06, respectively (Fig. 1B).

The above PCC values suggest that CD9 and EWI‐2 may only moderately associate with PIP_2_‐rich areas. However, it should be taken into account that PIP_2_ associates with many proteins, and that this large background would diminish specific PCCs of CD9 and EWI‐2, making comparisons difficult. Therefore, more specific PCC measurements in the absence of the aforementioned background are desirable. In the following, we allow for dissociation of PH, trying to trap the CD9‐proximal PH by antibody patching of CD9 via its GFP‐tag (Fig. S2). To this end, freshly prepared membrane sheets are incubated for 5 min (at room temperature) with a polyclonal anti‐GFP antibody, followed by fixation and incubation with a secondary antibody directed against the GFP antibody. The secondary antibody is not used for patching (it is added to fixed membranes) but to validate that the GFP antibody has bound to its target (Fig. S2, compare CD9‐GFP to secondary antibody image). The GFP‐antibody patching (αGFP) had a visible effect on the distribution of CD9, which is noticed in a better‐defined punctuate signal in comparison with the control (incubation without antibody), or to directly fixed membranes (compare respective green panels of Figs 2A, 4A, and Fig. S4A and C).

In the control without antibody, PH dissociation is essentially complete (Fig. 2B, compare directly fixed to control), whereas CD9 patching retains about 40% of PH (Fig. 2B, compare CD9 directly fixed to αGFP) and also increases the PCC between CD9 and PH (Fig. 2C). To exclude several types of artifacts, some control experiments were performed. First, we find that no PH is retained when CD9‐GFP is not expressed, indicating that the GFP antibody alone does not retain PH (Fig. S3B, compare control without antibody to αGFP_untr_). Second, despite CD9‐GFP expression, other polyclonal antibodies against EEA1 or EGFR do not retain PH (Fig. S3B) and also do not increase the PCC between CD9 and PH (Fig. S3C). These control experiments demonstrate that the antibody‐induced PH retainment and increase in PCC require the aggregation of CD9 by means of its GFP tag. Finally, antibody patching does not change the PCC between CD9‐GFP and CD81‐RFP or CD9‐GFP and CD151‐RFP (Fig. S4). Hence, the antibody treatment only patches the proteins but does not affect the degree of overlap. This suggests that patching, at least not within 5 min, does not largely affect the interaction network underlying the CD9‐CD81 and CD9‐CD151 association.

After the 5‐min period of PH dissociation, in the absence of most of the PH, the PCC between CD9 and PH does not change between directly fixed membranes and membranes incubated without antibody (Fig. 2C, compare control to directly fixed), but the PCC increases by several‐fold with the antibody (Fig. 2C; compare control to αGFP). This suggests the formation of CD9/antibody aggregates with therein trapped PH (Fig. S2, compare punctate signals of CD9‐GFP, secondary antibody and PH).

**Fig. 3:**
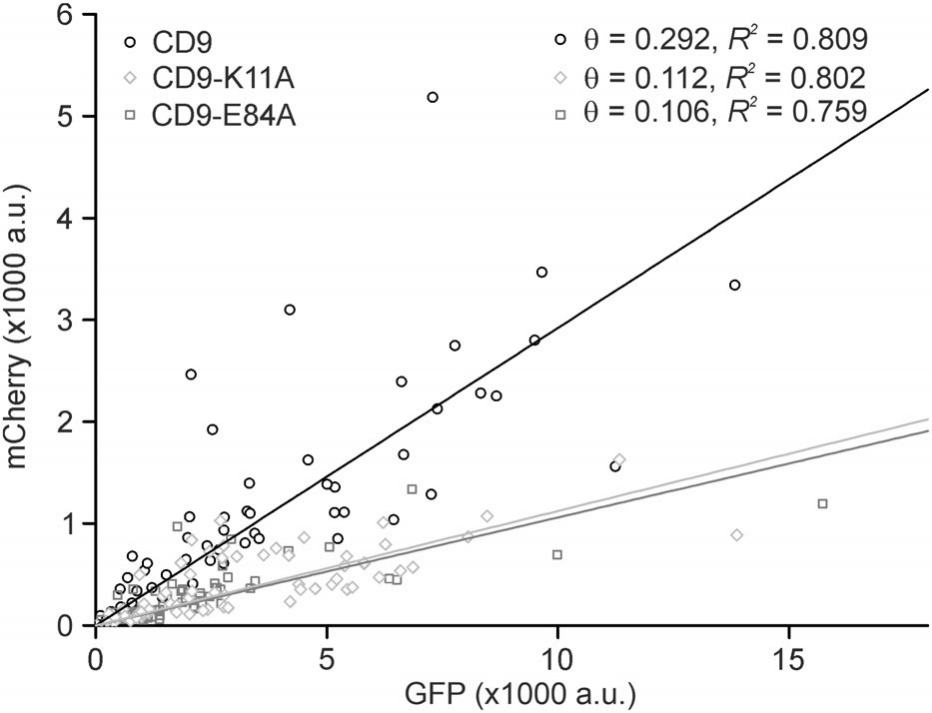
Relationship between retained PH and the expression level of GFP‐tagged CD9, CD9‐K11A, and CD9‐E84A. For individual membrane sheets analyzed in Fig. 2, condition αGFP, the intensity of mCherry (retained PH) is plotted versus the GFP intensity (expression level). For CD9, CD9‐K11A, and CD9‐E84A, the average GFP intensity was 4313 a.u. (55 membranes collected from 3 biological replicates), 2988 a.u. (58 membranes from 3 biological replicates) and 2375 a.u. (66 membranes from 3 biological replicates), respectively. Linear regression lines are fitted through the origin. *R* ^2^ values and slopes (θ) are stated in the upper right. Open circles, CD9; open diamonds, CD9‐K11A; open squares, CD9‐E84A.

In this assay, the open salt bridge mutants CD9‐K11A and CD9‐E84A retain significantly less PH than CD9 (Fig. 2B, inset), suggesting open‐CD9 is found less in PIP_2_‐rich areas/proximal to PIP_2_ than CD9 (the farther away PH is located from patched CD9, the less likely it becomes trapped). However, PH retainment should not exclusively depend on the proximity of PH to CD9, but on the abundancy of the patched protein too. In other words, the larger the area covered by the patched protein, the larger the PH fraction that becomes trapped. In Fig. 2, CD9 expression is stronger than CD9‐K11A or CD9‐E84A expression (see figure legend of Fig. 3). Therefore, CD9‐K11A and CD9‐E84A could be less effective in retaining PH just because of their lower abundancy. For a comparison that includes the expression level of the patched proteins, we plot the retained PH against GFP intensity for each membrane sheet. For all CD9 constructs, PH trapping increases with the expression level, but at the same GFP intensity, CD9 retains more PH when compared to CD9‐K11A or CD9‐E84A, while CD9‐K11A and CD9‐E84A are indistinguishable (Fig. 3). A decreased efficiency in PH retainment is reflected in a trend toward a smaller PCC between PH and CD9‐K11A or CD9‐E84A (Fig. 2C, αGFP, compare CD9 to CD9‐K11A and CD9‐E84A). Altogether, the data suggest that open‐CD9 is less proximal to PH. As PH reports on the lateral distribution of PIP_2_, we conclude that open‐CD9 is less associated with PIP_2_‐rich areas than CD9.

### Open‐CD9 and EWI‐2 prefer the same membrane regions

**Fig. 4:**
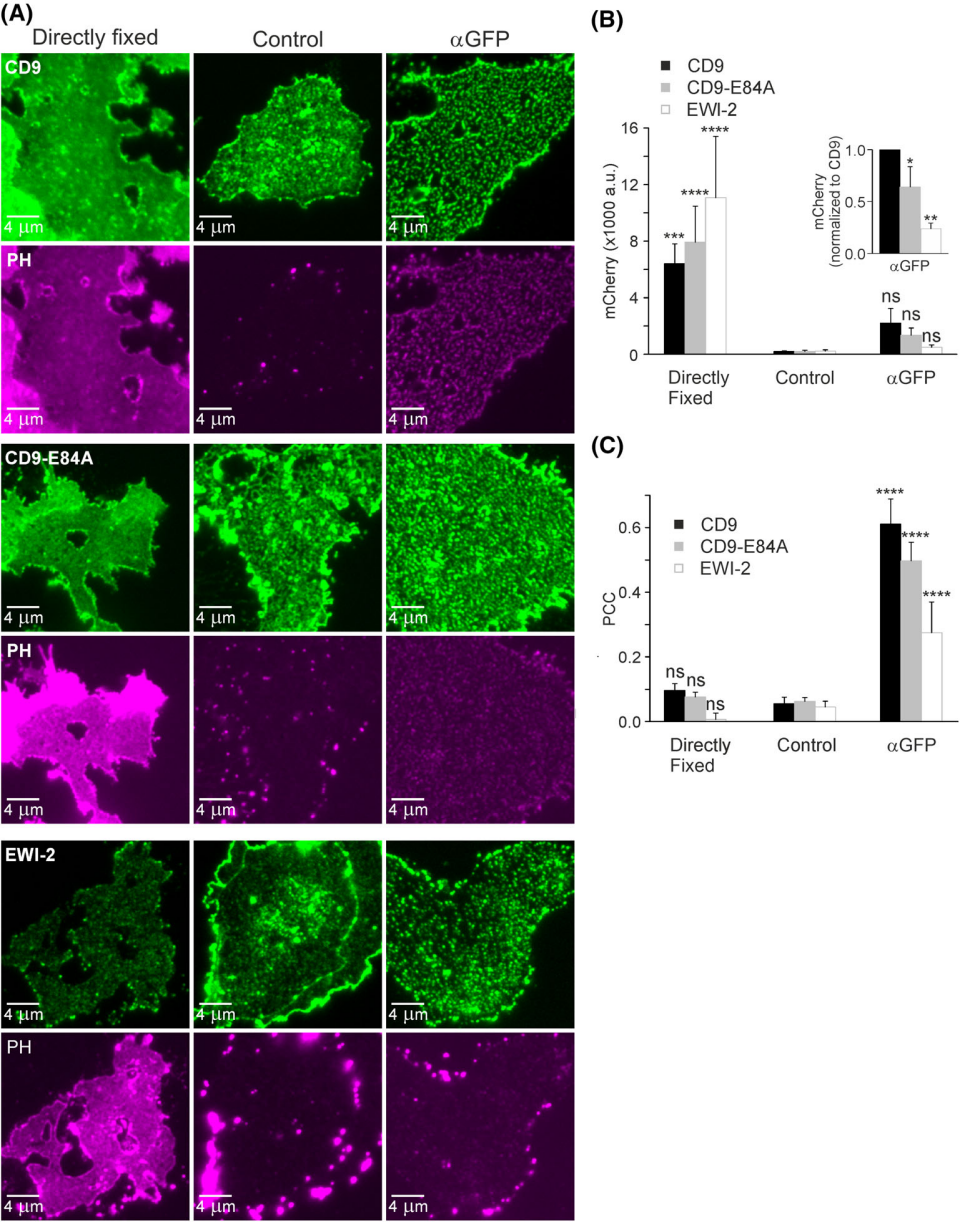
Antibody‐induced patching of CD9, CD9‐E84A, and EWI‐2. (A) HaCaT cells were double transfected with PH and GFP‐labelled CD9 (top), CD9‐E84A (middle), or EWI‐2 (bottom). One day after transfection, membrane sheets were generated and either directly fixed (left panels) or after a 5‐min incubation at RT without (Control, middle panels) or with an anti‐GFP antibody (αGFP, right panels). A green lookup table is used for CD9, CD9‐E84A, and EWI‐2, and a magenta lookup table for PH. Images of one channel are shown at the same settings of brightness and contrast. (B) The membrane‐associated PH (mCherry‐intensity) was analyzed with squared regions of interest (ROIs). The inset shows the condition αGFP after normalization of CD9‐E84A and EWI‐2 to CD9. (C) PCCs between PH and CD9, CD9‐E84A, and EWI‐2 were analyzed with squared ROIs. Values are given as means ± SD (*n* = 4 biological replicates; each replicate and condition includes 10–31 analyzed membrane sheets). Statistical test: two‐way ANOVA test followed by Šidák's multiple comparisons test comparing in (B) and (C) control to directly fixed or αGFP (ns: non‐significant; ***P < 0.001; ****P < 0.0001). (B) Inset, the confidence interval was calculated for the comparison of CD9‐E84A to CD9 (*P < 0.05) or EWI‐2 to CD9 (**P < 0.01).

**Fig. 5:**
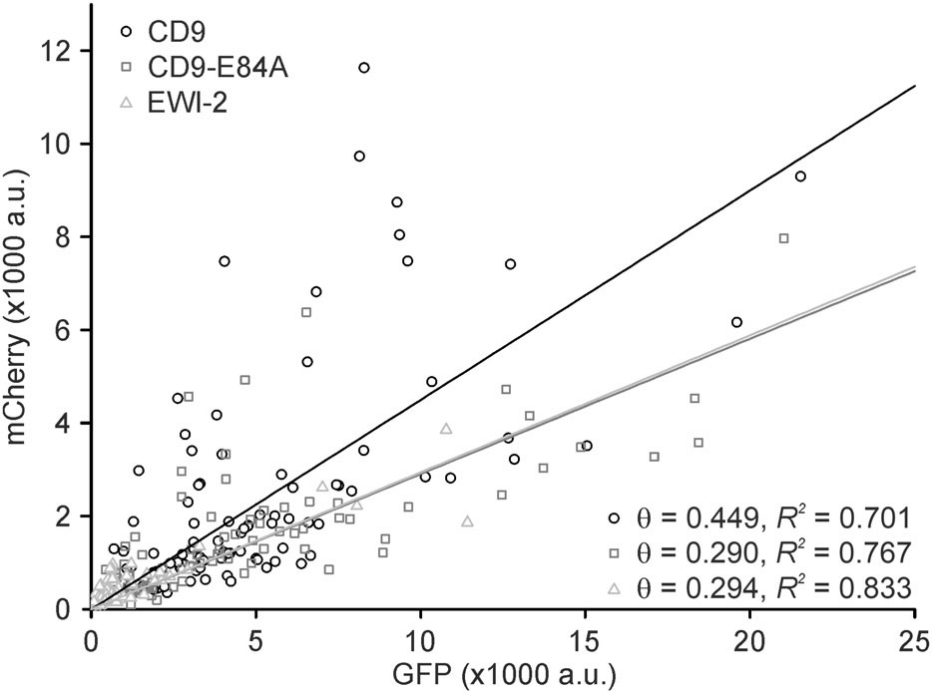
Relationship between retained PH and the expression level of GFP‐tagged CD9, CD9‐E84A, and EWI‐2. For individual membrane sheets analyzed in Fig. 4 (condition αGFP), the intensity of mCherry (retained PH) is plotted versus the GFP intensity (expression level). For CD9, CD9‐E84A, and EWI‐2, the average GFP intensity was 4865 a.u. (96 membranes collected from 4 biological replicates), 4278 a.u. (93 membranes from 4 biological replicates), and 1269 a.u. (66 membranes from 4 biological replicates), respectively. Regression lines are fitted through the origin. *R* ^2^ values and slopes (θ) are stated in the lower right. Open circles, CD9; open squares, CD9‐E84A; open triangles, EWI‐2.

Next, we compared CD9, CD9‐E84A, and EWI‐2 (Fig. 4A). Again, CD9‐E84A retains less PH than CD9 (Fig. 4B, inset). Patching of EWI‐2 increases significantly the PCC between EWI‐2 and PH to 0.28 (Fig. 4C, compare control to αGFP) and tends to retain some PH (Fig. 4B, compare control to αGFP). However, the fraction of retained PH is quite small, possibly because EWI‐2 expression is much lower than CD9 or CD9‐E84A expression (for figures see legend of Fig. 5). Plotting PH retainment against expression level reveals that EWI‐2 is actually indistinguishable from CD9‐E84A (Fig. 5). Altogether, the patching assay suggests again that CD9 associates stronger with PIP_2_ areas than the open salt bridge variant, and that EWI‐2 behaves like open‐CD9.

**Fig. 6:**
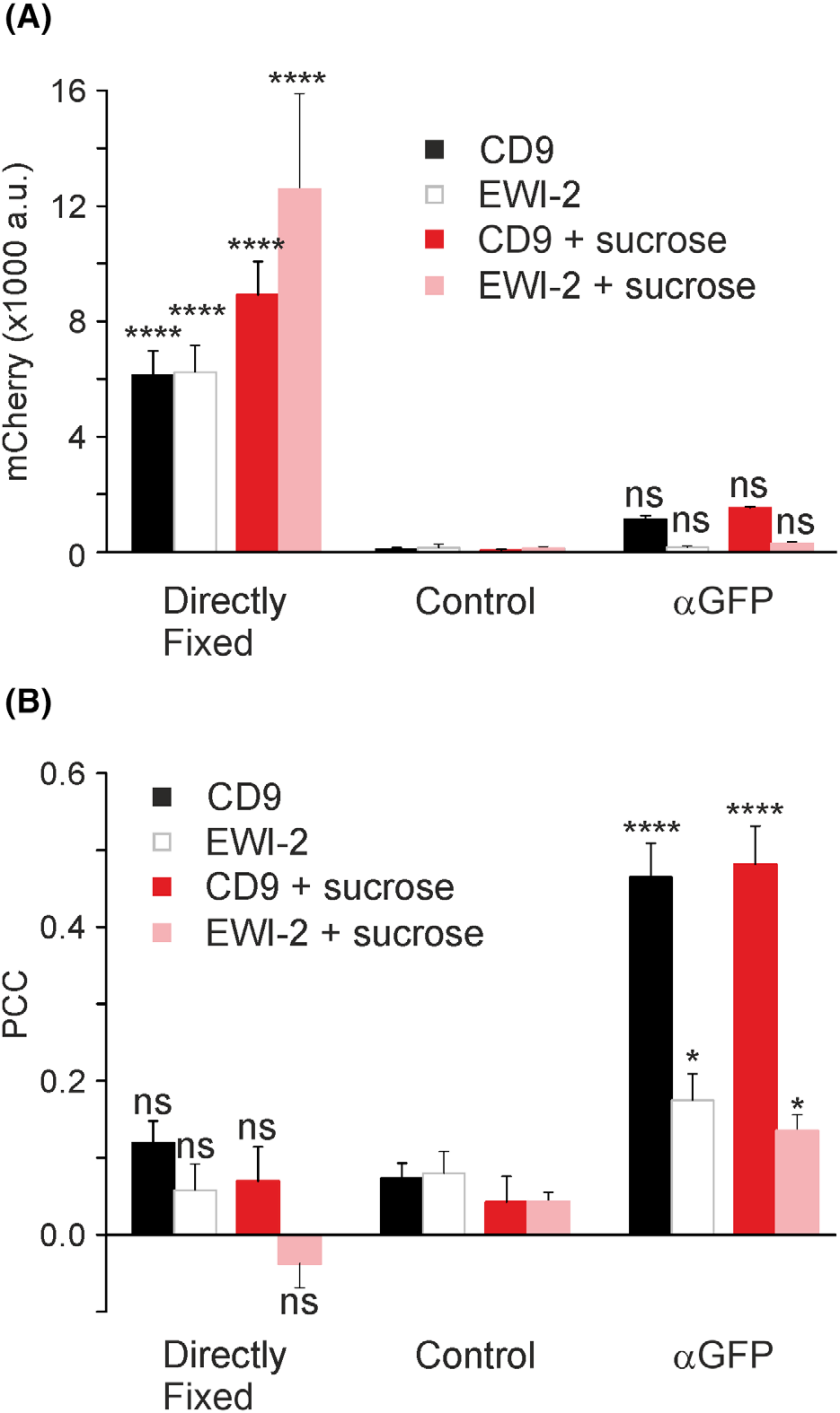
Antibody‐induced patching of CD9 and EWI‐2 after elevation of PIP_2_. HaCaT cells were double transfected with PH and GFP‐labelled CD9 or EWI‐2, and one day after transfection, membrane sheets were generated. When indicated, prior to membrane sheet generation, cells were incubated for 15 min with 300 mm sucrose. Then, cells were either directly fixed or after a 5‐min incubation at RT without (Control) or with an anti‐GFP antibody (αGFP). (A) The average membrane‐associated PH (mCherry‐intensity) analyzed with squared regions of interest (ROIs). (B) PCCs were analyzed between PH and CD9 or EWI‐2 with squared ROIs. Values are given as means ± SD (*n* = 3 biological replicates; each replicate and condition includes 12–43 analyzed membrane sheets). Statistical test: two‐way ANOVA test followed by Šidák's multiple comparisons test comparing in (A) and (B) control to directly fixed or αGFP (ns: non‐significant; *P < 0.05; ****P < 0.0001).

Subsequently, we wondered whether the difference between CD9 and EWI‐2 may be abolished under elevated PIP_2_ levels. PIP_2_ was elevated by a 15 min treatment with 300 mM sucrose that has been shown to double the amount of plasmalemmal PIP_2_ [31]. In line with the previous report, we find with 300 mm sucrose an up to 100% increase of the PIP_2_‐reporter PH bound to the cell membrane (Fig. 6A, directly fixed, compare EWI‐2 without and with sucrose). Also under elevated PIP_2_, CD9 is more effective in PH retainment than EWI‐2 (Fig. 6A, αGFP, compare CD9 and EWI‐2 without and with sucrose). Similar to Figs 2C and 4C, the PCC between patched CD9 and retained PH was several‐fold increased, whereas the increase in the PCC between EWI‐2 and PH is smaller (Fig. 6B, compare control to αGFP). Hence, the PIP_2_ level of the cell membrane has no measurable effect on the different preferences of CD9 and EWI‐2 for PIP_2_‐rich membrane areas.

**Fig. 7:**
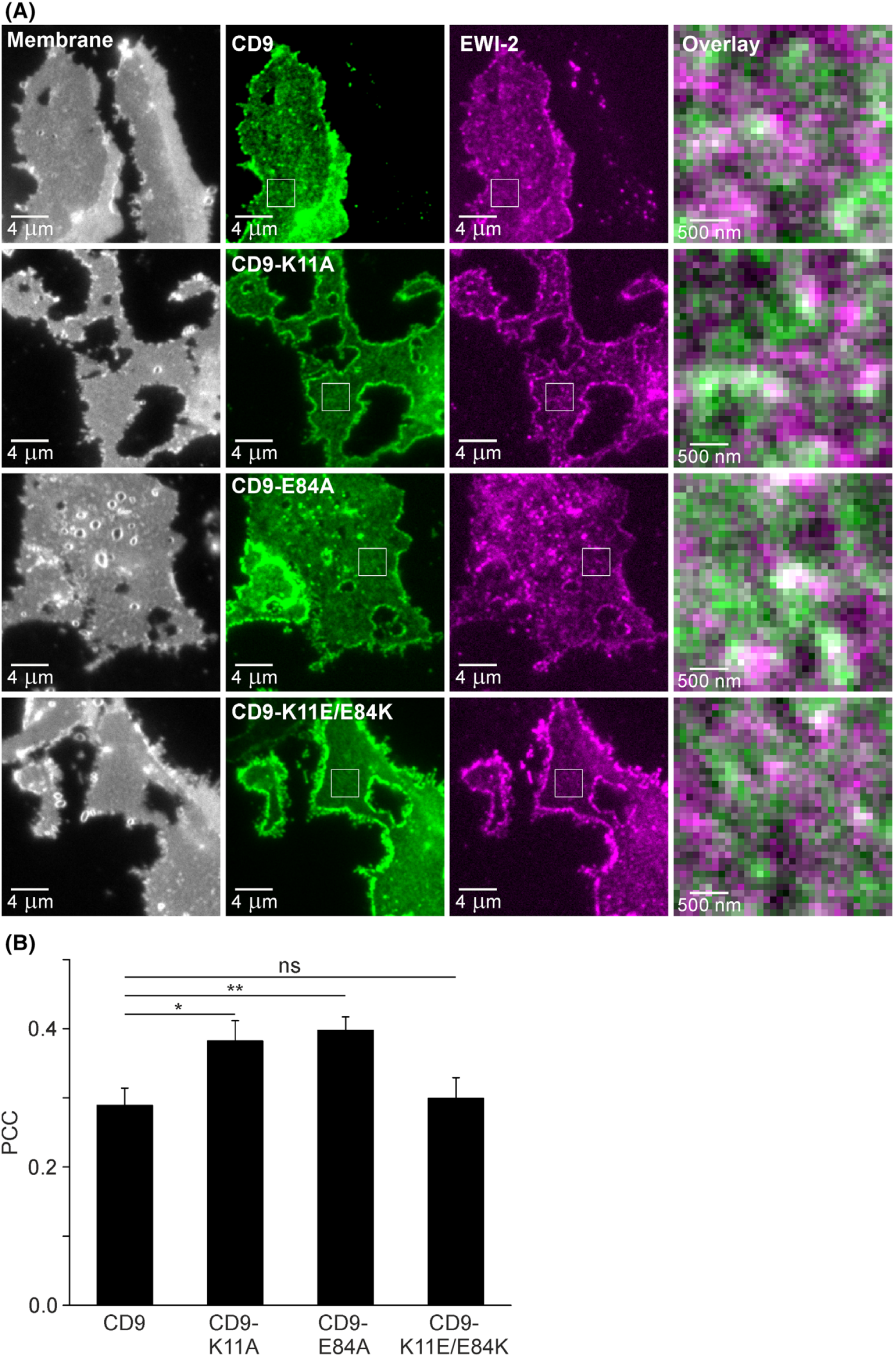
Pearson correlation coefficient (PCC) between EWI‐2 and CD9, CD9‐K11A, CD9‐E84A, or CD9‐K11E/E84K analyzed on directly fixed basal membrane sheets. (A) HaCaT cells were double transfected with EWI‐2 (fused to monomeric RFP) and CD9, CD9‐K11A, CD9‐E84A, or CD9‐K11E/E84K (each fused to monomeric GFP). One day after transfection, membrane sheets were generated, fixed, and imaged. Left column, visualization of membranes by the dye TMA‐DPH (displayed with a grayscale lookup table); middle columns, CD9, CD9‐K11A, CD9‐E84A, or CD9‐K11E/E84K (green lookup table) and EWI‐2 (magenta lookup table); right column, magnified views show overlays from the boxed regions of the middle images. With the exception of the TMA‐DPH images (left column), images of one channel are shown at the same settings of brightness and contrast. (B) PCC between EWI‐2 and CD9, CD9‐K11A, CD9‐E84A, or CD9‐K11E/E84K analyzed with squared regions of interest (ROIs). Values are given as means ± SD (*n* = 3 biological replicates; each replicate and condition includes 14–29 analyzed membrane sheets). Statistical test: one‐way ANOVA test followed by Dunnett's multiple comparisons test comparing in (B) CD9 to CD9‐K11A, CD9‐E84A, and CD9‐K11E/E84K (ns: non‐significant; *P < 0.05; **P < 0.01).

The observation that the open‐CD9 variants and EWI‐2 associate less with PIP_2_ areas than CD9 does not necessarily imply that open‐CD9 and EWI‐2 populate the same membrane regions. For clarification, we analyzed the overlap of all the CD9 variants with EWI‐2 (Fig. 7A). We find that elimination of the salt bridge (CD9‐K11A and CD9‐E84A) significantly increases the overlap with EWI‐2, whereas the mutant with interchanged charges (CD9‐K11E/E84K) is indistinguishable from wild‐type CD9 (Fig. 7B). In a previous co‐immunoprecipitation experiment, open‐CD9 increases the pull‐down of EWI‐2 by up to 50%, whereas the interchange of salt bridge charges has no effect [16]. Hence, the association between CD9 and EWI‐2, either measured by microscopic overlap on membrane sheets or by pull‐down after cell solubilization, increases upon the salt bridge opening.

### Effect of the salt bridge on the association of CD9 with CD9

**Fig. 8:**
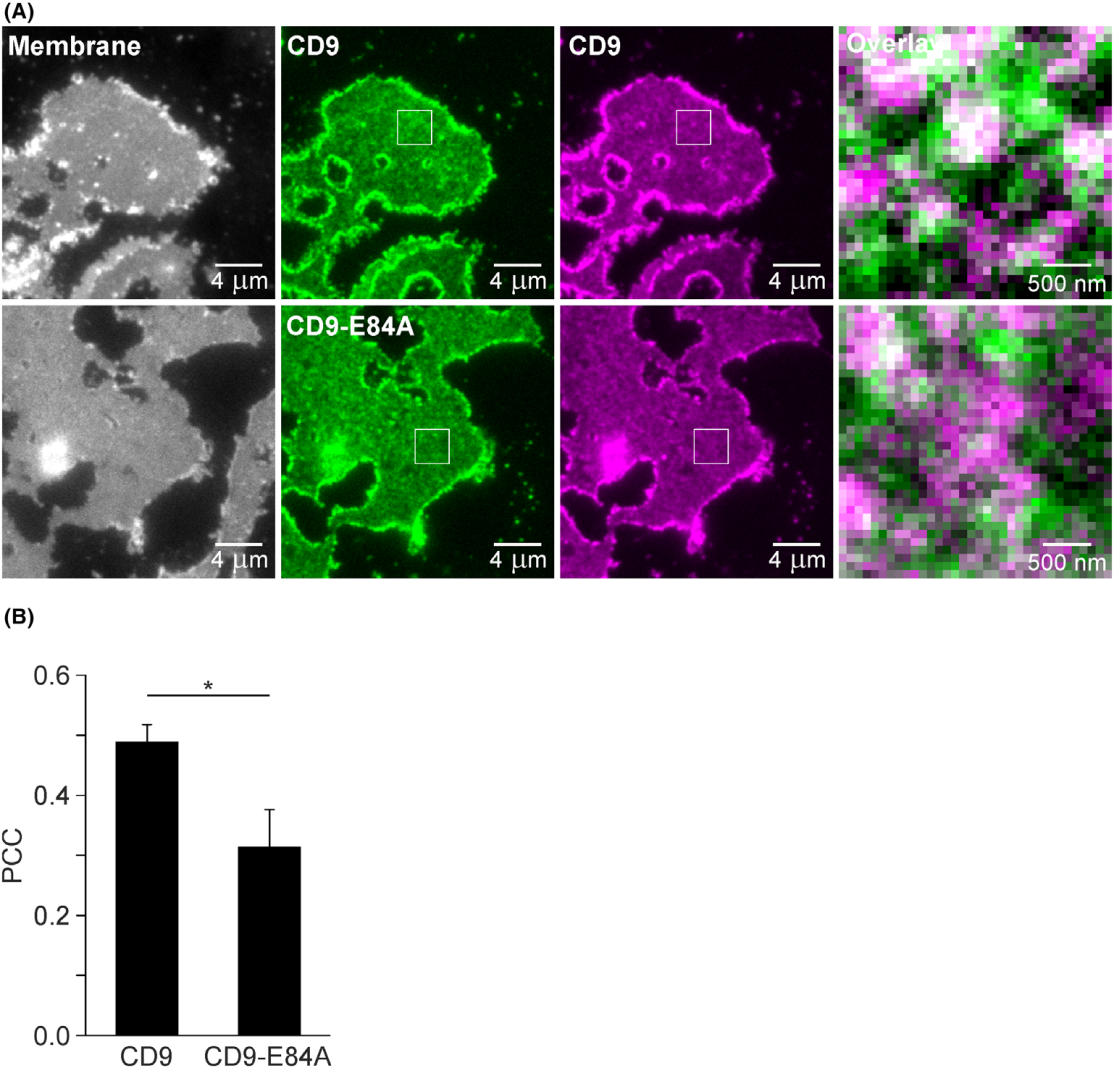
Pearson correlation coefficient (PCC) between CD9‐RFP and CD9‐GFP or CD9‐E84A‐GFP. (A) HaCaT cells were double transfected with RFP‐labelled CD9 and GFP‐labelled CD9 or CD9‐E84A. One day after transfection, membrane sheets were generated, fixed, and imaged. Left, visualization of membranes by TMA‐DPH (grayscale lookup table); two middle columns, GFP‐labelled CD9 or CD9‐E84A (left, green lookup table) and RFP‐labelled CD9 (right, magenta lookup table); right, magnified views show overlays from the boxed regions of the middle images. With the exception of the TMA‐DPH images (left), images of one channel are shown at the same settings of brightness and contrast. (B) PCC between RFP‐labelled CD9 and GFP‐labelled CD9 or CD9‐E84A analyzed with squared regions of interest (ROIs). Values are given as means ± SD (*n* = 3 biological replicates; each replicate and condition includes 23–31 analyzed membrane sheets). Statistical test: unpaired two‐tailed Student's *t*‐test (*P < 0.05).

In the following, we study the association of RFP‐labelled CD9 with GFP‐labelled CD9 or CD9‐E84A (Fig. 8A). The PCC between CD9‐RFP and CD9‐GFP is significantly larger when compared to CD9‐RFP and CD9‐E84A‐GFP (Fig. 8B), suggesting that, as already expected from the patching assay, CD9 and open‐CD9 prefer different membrane areas.

**Fig. 9:**
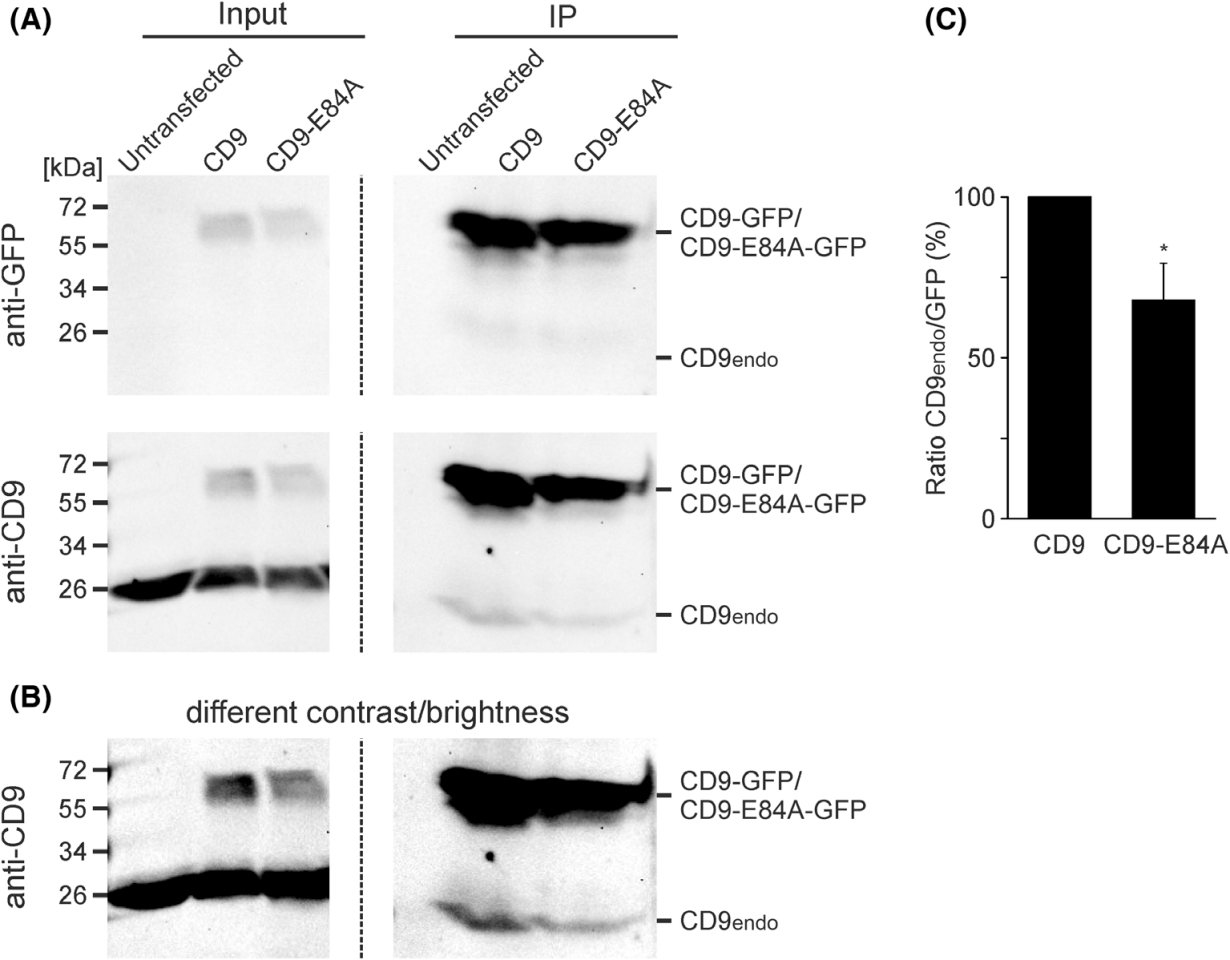
Opening of CD9 diminishes co‐immunoprecipitated endogenous CD9. (A) HaCaT cells were transfected with GFP‐tagged CD9 or CD9‐E84A. One day after transfection, cells were lysed in 1% Brij97 (input shows the lysate) and the GFP‐tag was pulled down via GFP‐trap beads (IP, immunoprecipitate). Untransfected cells were used as control. Proteins were separated by electrophoresis (SDS‐PAGE), transferred onto a nitrocellulose membrane, followed by the detection of CD9 (lower membrane) and GFP (upper membranes). CD9‐ and GFP western blots are shown at different settings of brightness and contrast. Left and right images (separated by a dashed line) are from the same membrane and shown at the same brightness and contrast settings. Please note that on the lower membrane both overexpressed and endogenous CD9 are detected by the CD9 antibody, whereas on the upper membrane, only the overexpressed GFP‐tagged CD9/CD9‐E84A is detected. (B) The lower membrane in (A) is shown at a different brightness and contrast setting for better illustration of the difference in the bands of co‐precipitated endogenous CD9. (C) The immunoprecipitation efficiency is determined from the IP membranes. Co‐immunoprecipitated endogenous CD9 (CD9‐antibody detection, lower CD9endo band) is related to the band of the respective immunoprecipitated GFP (GFP‐antibody detection, CD9‐GFP/CD9‐E84A‐GFP). For each replicate, the ratio of CD9endo/CD9‐GFP and CD9endo/CD9‐E84A‐GFP was calculated and normalized to CD9endo/CD9‐GFP (set to 100%). Values are given as means ± SD (*n* = 4 biological replicates). Statistical test: (C) the confidence interval was calculated for the comparison of CD9‐E84A to CD9 (*P < 0.05).

The effect of the salt bridge on CD9‐CD9 association is also assayed in immunoprecipitation experiments (Fig. 9A). Cells express either GFP‐tagged CD9 or CD9‐E84A, and we probe for the interaction of the overexpressed proteins with endogenous CD9. After solubilization with 1% Brij97, we pull down the overexpressed proteins via the GFP tag and quantify co‐immunoprecipitated endogenous CD9. As shown in Fig. 9C, open‐CD9 co‐precipitates almost a third less endogenous CD9 than wild‐type CD9.

**Fig. 10:**
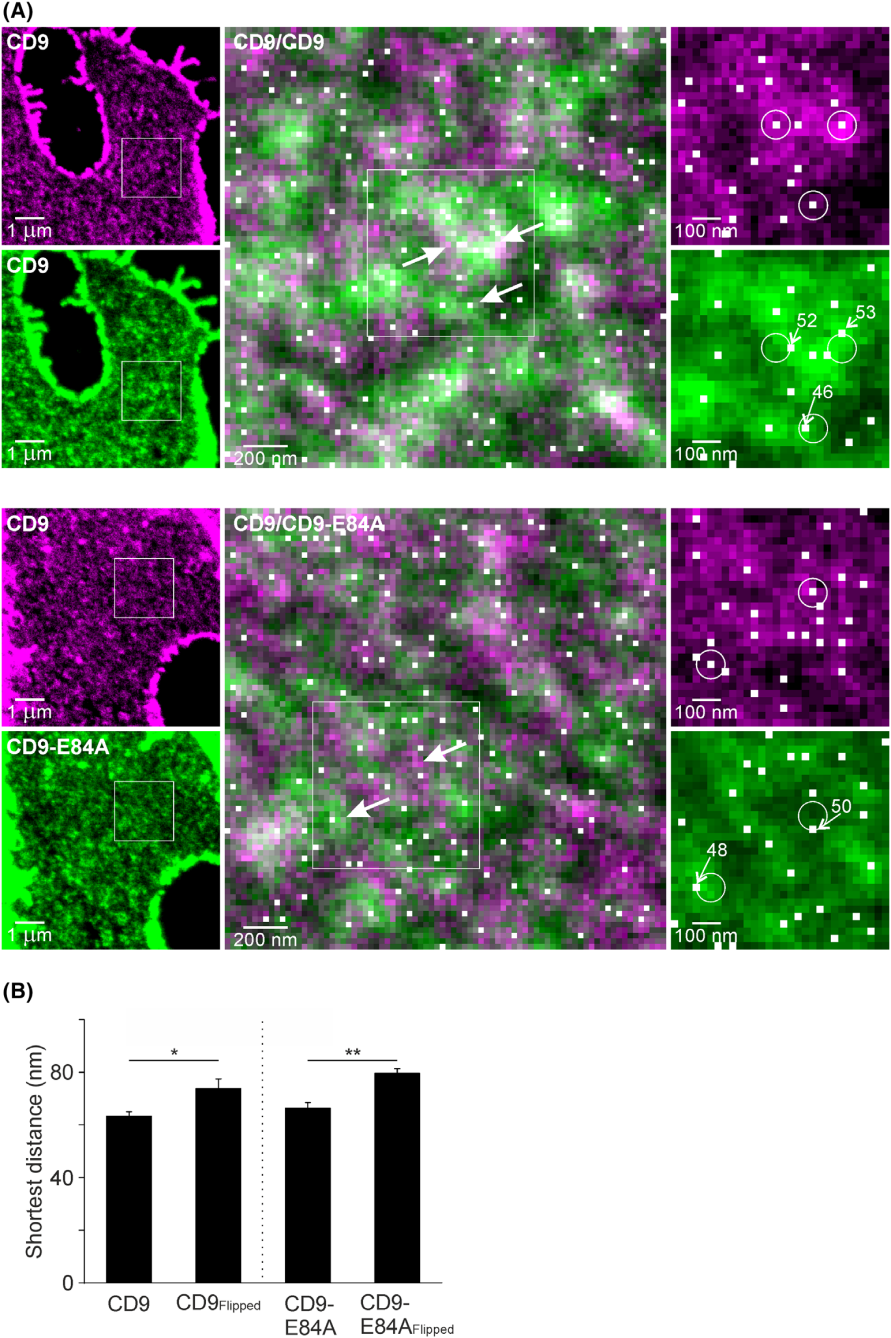
CD9 opening does not change the distance of CD9 to the next neighbored CD9. (A) After the imaging of membrane sheets by epi‐fluorescence microscopy (Fig. 8), membranes were incubated with GFP and RFP nanobodies coupled to dyes enabling super‐resolution imaging by STED microscopy. Magenta and green images show STED micrographs recording the distribution of CD9‐RFP and CD9‐GFP/CD9‐E84A‐GFP, respectively. Middle images are overlays of magnified views of the boxed regions in the left images. Right, magnified views of the boxed regions of the middle images. Maxima are detected in the CD9‐RFP channel (white pixels in the right magenta images) and classified as nano‐clusters (encircled white pixels; for details see text). For each nano‐cluster in the magenta image, the distance to the next nearest maximum in the green image (white pixels mark all maxima) was determined. Numbers in the right green images state the determined shortest distances in nanometers. Middle, white pixels mark maxima of the green image, and arrows mark the positions of the encircled nano‐clusters in the magenta images. (B) Average shortest distance of CD9 to CD9 (left) or CD9 to CD9‐E84A (right), compared with the shortest distance in flipped images (used as control for obtaining the purely random distance). Values are given as means ± SD (*n* = 3 biological replicates; each replicate and condition includes 10–17 analyzed membrane sheets). Statistical test: unpaired two‐tailed Student's *t*‐test between CD9 and CD9_Flipped_ (left, *P < 0.05) or CD9‐E84A and CD9‐E84A_Flipped_ (right, **P < 0.01).

Employing super‐resolution microscopy, several studies have shown that tetraspanins concentrate in nano‐clusters with diameters in the range of 100 nm [32, 33], which contain several tetraspanin copies [32]. As described for other membrane proteins [34], also in our images the nano‐clusters are not evenly distributed, but gather together in strongly populated areas surrounded by regions without nano‐clusters (see dark zones in the overlays in Fig. 10A). Conventional microscopy does not readily resolve these nano‐clusters. Therefore, so far we have analyzed overlap of areas whose shapes are generated by merged nano‐cluster signals blurred by the point spread function of the microscope. In Fig. 8, reduction in the PCC between CD9‐RFP and CD9‐E84A‐GFP suggests transfer of open‐CD9 molecules to areas with less PIP_2_. However, we do not know whether entire nano‐clusters or single molecules translocate. The following two extreme scenarios are possible. First, CD9 and open‐CD9 form completely separated nano‐clusters. This implies that we never find an open‐CD9 molecule in a CD9 nano‐cluster, and the other way around. In the second scenario, mixed clusters form. If a nano‐cluster forms in a PIP_2_‐rich areas, because here CD9 is more abundant, it will contain more CD9 than open‐CD9 molecules, whereas in the low PIP_2_ areas, it is the other way around. In any case, in a PIP_2_‐rich area, any CD9 nano‐cluster is close to an open‐CD9 molecule, either because it contains one, or an open‐CD9 molecule is present in the next neighbored CD9 nano‐cluster. The distance from CD9 nano‐clusters to the next CD9 or open‐CD9 molecule is similar. In contrast, if CD9 and open‐CD9 segregate into different nano‐clusters, CD9 nano‐clusters in the center of PIP_2_ areas have a short distance to the next neighbored CD9 molecule, but a rather long distance to the next open‐CD9 molecule present in a neighboring area. Hence, if CD9 and open‐CD9 segregate into different nano‐clusters, the shortest distance between CD9 and open‐CD9 should be larger than the distance between CD9 and CD9.

For addressing this question, we employed super‐resolution STED microscopy. We used the same samples already recorded for Fig. 8. However, as GFP and RFP are not suitable for STED microscopy, we incubated the fixed membranes with GFP/RFP nanobodies coupled to suitable dyes. As shown in Fig. S5, the GFP pattern is reproduced by the GFP nanobody, although images are not entirely congruent. When compared to diffraction‐limited microscopy, STED microscopy resolves the signal into sharper spots (Fig. S6). The analysis detects local maxima in the CD9 channel (Fig. 10A, see white pixels in the right magenta images). To reduce noise, we only include these maxima if they are well defined (see encircled white pixels in the right magenta images). For classification, we place linescans onto the maxima and fit Gaussian functions to the recorded intensity traces. If the fit quality of the Gaussian is > 0.7 and if the Gaussian maximum lies in the middle third of the 31‐pixel linescan, we rate the maximum as well defined and classify it as a nano‐cluster. Then, we determine the shortest distance of these nano‐clusters to a maximum in the other channel (Fig. 10A, see white pixels in the right green images, values state the distance in nm). As a randomized control, the same analysis is performed after flipping the image of one channel. The shortest distance at random, which depends on the maxima density, is 74 nm and 80 nm for CD9 to CD9 (Fig. 10B, CD9_Flipped_) and CD9 to CD9‐E84A (Fig. 10B, CD9‐E84A_Flipped_), respectively. In the original images, the distances are significantly smaller, around 65 nm (Fig. 10B), but we do not find an increase in the shortest distance between CD9 and CD9‐E84A. The analysis was repeated with three more noise tolerance settings for maxima detection (see Materials and methods for details). In summary, the larger the noise tolerance, the fewer maxima are detected, which increases the shortest distances. However, there are no settings that result in a change in the shortest distance, suggesting that CD9 and open‐CD9 form rather mixed than strictly separated nano‐clusters.

## Discussion

### Expression level of open‐CD9

In a previous study, we examined whether elimination of the salt bridge affects the trafficking of CD9 in HepG2 cells. Confocal micrographs from the cells' equatorial plane showed that CD9 and open‐CD9 overlap with an ER‐marker equally well, suggesting no major missorting into, for example, lysosomes. Moreover, in western blots, in comparison with CD9, open‐CD9 expression was halved [16].

In HaCaT cells, in western blot experiments (Fig. 9), we find only a small reduction (11%) of the CD9‐E84A‐GFP band when compared to CD9. In micrographs of directly fixed membranes of Fig. 2, compared with CD9, the GFP signal of CD9‐K11A or CD9‐E84A was 11–22% smaller, and reduced by 31–45% on patched membranes (see legend of Fig. 3). Hence, also in HaCaT cells, open‐CD9 expression is diminished. To exclude that different expression levels underlie our findings, we relate PH retainment to expression level in the patching experiments (Figs 3 and 5), and in the western blot experiment pulled‐down endogenous CD9 is related to overexpressed CD9‐GFP or CD9‐E84A‐GFP. Finally, altered expression levels do not explain changes in the PCCs (Figs 1, 2, 4, 6, 7 and 8), as the PCC is independent of the absolute value of the signal intensity.

### The spatial distribution of CD9 is regulated by the salt bridge

The question addressed in this study is whether the CD9 salt bridge is possibly regulating the proximity of CD9 to the second messenger PIP_2_, what in turn may have a regulatory effect on the CD9‐EWI‐2 interaction. For addressing this question, we studied membrane sheets by microscopy, comparing relative to PH the distribution of CD9, open‐CD9 (CD9‐K11A and CD9‐E84A) and EWI‐2. In these experiments, PH is used as a reporter of PIP_2_ distribution [29]. As PIP_2_ is highly abundant in the membrane, PH binds to many proteins, causing strong background PH‐fluorescence in addition to the CD9‐/EWI‐2‐specific PH‐fluorescence. In order to eliminate this background, we washed off PH while trapping PH proximal to GFP‐tagged CD9 or EWI‐2. Similar to PIP_2_‐bound PH, GFP is near to the intracellular membrane leaflet, as it is fused to a 9 and 13 amino acid intracellular peptide of CD9 and EWI‐2, respectively. This is an ideal situation for efficient trapping. Hence, the polyclonal antibody against GFP aggregates CD9‐GFP molecules, and if PH is in physical proximity to these molecules, it becomes trapped in the forming aggregates (see also Fig. S2).

The patching process does not change the PCC between CD9 and CD81 or CD9 and CD151 (Fig. S4), but significantly increases by several‐fold the PCC between PH and CD9, CD9‐K11A, CD9‐E84A, and EWI‐2 (Figs 2C, 4C and 6B). Moreover, it prevents PH wash off (insets in Figs 2B and 4B). Because the PCC is independent of the absolute values of signal intensity, we propose PH retainment is better suited for relatively comparing whether proteins are proximal to PH, or in other words, whether they associate with areas rich in PIP_2_. We assume the method is only semi‐quantitative, which is why we can only compare pairs of proteins. For CD9, we observe the strongest retainment and largest PCC values, whereas the two open‐CD9s and EWI‐2 associate less with PH (insets in Figs 2B and 4B; Figs 2C, 4C, 6A and 6B) and show no difference to each other in pairwise comparisons (CD9‐K11A and CD9‐E84A are equal in Fig. 3, and CD9‐E84A and EWI‐2 are equal in Fig. 5). CD9 patching is capable of retaining 34–42% of PH (Figs 2B and 4B), which to our understanding is a large figure considering that (i) CD9 is only one out of many proteins that possibly associates with PIP_2_ areas and (ii) most likely not all PH proximal to CD9‐GFP is trapped. Therefore, we propose that CD9 locates in areas that are enriched in PIP_2_, in contrast to open‐CD9 and EWI‐2.

If CD9 associates with PIP_2_ areas via electrostatic interactions involving the N‐terminal lysines K4, K8, and K11, open‐CD9‐E84A should associate more strongly with PIP_2_ areas because (i) the positive charge of K11 is no longer neutralized in the salt bridge (increasing the total positive charge), and (ii) the N‐terminus is no longer tightly attached to the SIL, which should increase the conformational flexibility for PIP_2_ binding. The latter argument is also valid regarding open‐CD9‐K11A. However, the open‐CD9 variants do not associate more strongly but less with PIP_2_ areas. Therefore, we consider electrostatic binding to PIP_2_‐rich areas as a type of recruiting mechanism rather unlikely and propose that a conformational change is responsible for the transfer from one area to another.

**Fig. 11:**
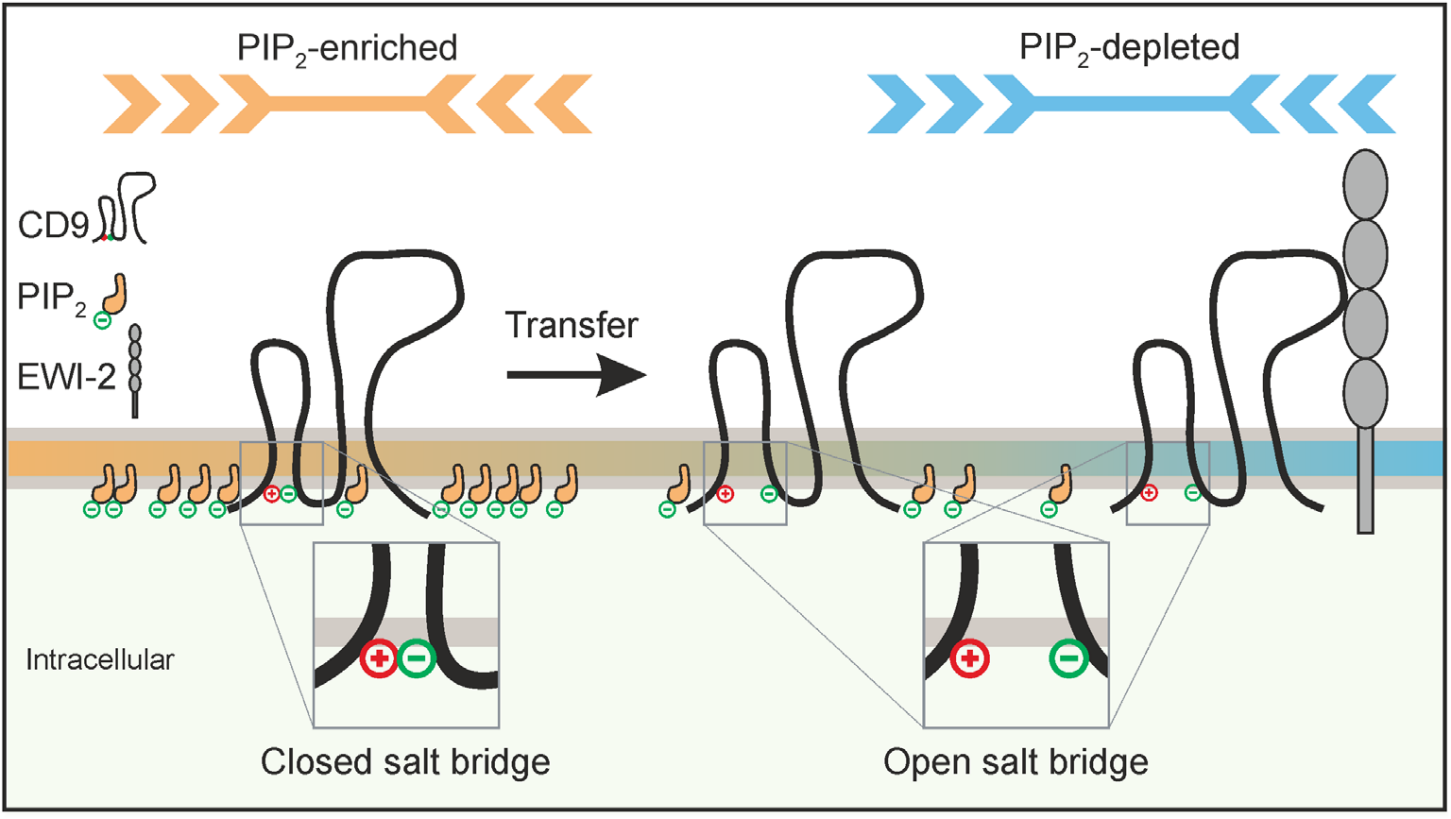
CD9 distribution in the membrane depends on the state of the salt bridge. In CD9, a salt bridge forms between a negatively charged glutamate of the SIL and a positively charged lysine of the N‐terminus [16] (see magnified view of the closed salt bridge; the red circle with a plus sign and the green circle with a minus sign represent a lysine and a glutamate residue, respectively). In the closed conformation, CD9 prefers PIP_2_‐enriched areas. In contrast, CD9 lacking the salt bridge prefers areas in which PIP_2_ is depleted, like the CD9‐interaction partner EWI‐2. A transfer of open‐CD9 to PIP_2_‐depleted areas would promote the complex formation between CD9 and EWI‐2.

In conclusion, the salt bridge regulates the association of CD9 with PIP_2_‐rich areas, and open‐CD9 locates in areas that are less enriched in PIP_2_ but more strongly populated with EWI‐2 (Fig. 11).

### Effect of the salt bridge on homo‐oligomerization

In the immunoprecipitation experiment, open‐CD9 co‐precipitates a third less endogenous CD9 (Fig. 9C), indicating less CD9‐homo‐oligomers forming between endogenous CD9 and open‐CD9 than with CD9. In microscopy, open‐CD9‐GFP overlaps less with CD9‐RFP than CD9‐GFP (Fig. 8B). This suggests that open‐CD9‐GFP and endogenous CD9 prefer different membrane areas. In this scenario, the diminished co‐precipitation may not be explained by a diminished affinity between open‐CD9 and CD9, but rather by the spatial separation of endogenous CD9 and open‐CD9 that produces fewer encounters between the molecules. Therefore, we consider it as likely that the salt bridge has no effect on the homo‐oligomerization affinity. Regarding the shortest distance between CD9 to CD9 and CD9 to CD9‐E84A, we also observe no salt bridge effect, suggesting that the salt bridge does not affect CD9 nano‐clustering. However, in this experiment, super‐resolution STED microscopy may have reached its limit; wherefore we cannot exclude that with a higher resolution and a more efficient nanobody labelling a difference would have been revealed. Altogether, the current data suggest that the opening of the salt bridge generates a CD9 conformation with a lower preference for PIP_2_‐rich areas, and rather does not directly affect homo‐oligomerization and nano‐clustering.

### A mechanism explaining the inhibitory effect of the salt bridge on CD9‐EWI‐2 association

Based on our patching analysis, we assign CD9 to PIP_2_‐rich areas, and open‐CD9/EWI‐2 to areas with less PIP_2_. Microscopic analysis on directly fixed membranes further shows that open‐CD9 overlaps better with EWI‐2 (Fig. 7). In co‐immunoprecipitation experiments, the CD9‐K11A and CD9‐E84A mutations increase CD9‐EWI‐2 association but not CD9‐K11E/E84K [16].

All findings can be combined to a coherent picture: CD9 has a stronger preference for PIP_2_‐rich areas than open‐CD9 and EWI‐2, whereas open‐CD9 and EWI‐2 prefer the remaining regions. Hence, open‐CD9 transfers to regions also preferred by EWI‐2, where it encounters EWI‐2 more frequently (Fig. 11). Under the assumption that the reaction rate is proportional to the collision frequency, more CD9‐EWI‐2 complexes do form, explaining how the salt bridge regulates the CD9‐EWI‐2 interaction.

At present, we do not know what fraction of wild‐type CD9 is in the closed‐state. However, if all CD9 molecules would adopt an open conformation, we should not find any effect of the open mutants. Therefore, we propose that some CD9 adopts the closed conformation.

### Future perspectives

It is tempting to speculate that tetraspanins are capable of sensing intracellular messengers in a direct fashion. Here, we show that disabling of the salt bridge between the SIL and the N‐terminus weakens the association of CD9 with PIP_2_ areas, which is accompanied by an increase in CD9‐EWI‐2 association. The far‐reaching question is whether PIP_2_ levels changing during signaling may regulate tetraspanins by changing their lateral distribution via this process. Moreover, PIP_2_ binding to K11 and the closed salt bridge are mutually exclusive. CD9 may be opened by a rise in PIP_2_, promoting PIP_2_ binding to K11. This would be enforcing the open conformation and trigger CD9 movement to EWI‐2 regions. These and many other questions remain open. Our study is just a first step toward addressing the topic of molecular crosstalk between tetraspanins and second messengers, which is, as we believe, an important issue for understanding how this enigmatic protein family is able to regulate so many functions.

## Conflict of interest

The authors declare no conflict of interest.

## Authors contributions

YH and SCK performed the experiments and analyzed the data. YH and TL designed the experiments. YH and TL wrote the manuscript.

## Supporting information


**Fig. S1.** Illustration of the reversible binding of PH to PIP_2_.
**Fig. S2.** PH trapping by GFP‐antibody‐induced CD9‐GFP‐aggregation.
**Fig. S3.** Antibody‐patching control experiments.
**Fig. S4.** Antibody‐induced CD9 patching does not affect the PCC between CD9 and CD81 or CD9 and CD151.
**Fig. S5.** Overlap between CD9‐GFP and the nanobody labelling of CD9‐GFP.
**Fig. S6.** Difference between diffraction limited and super‐resolution STED microscopy.

## Data Availability

Data of the current study are available from the corresponding author upon reasonable request.
