# Supplementary material for: CD9‐association with PIP2 areas is regulated by a CD9 salt bridge
Source: FEBS Open Bio. 2025 Jul 18;15(10):1678–95. doi: 10.1002/2211-5463.70084 (PMC12485883; doi:10.1002/2211-5463.70084)
Supplement: Supplementary file 1 — Fig. S1. Illustration of the reversible binding of PH to PIP2. Fig. S2. PH trapping by GFP‐antibody‐induced CD9‐GFP‐aggregation. Fig. S3. Antibody‐patching control experiments. Fig. S4. Antibody‐induced CD9 patching does not affect the PCC between CD9 and CD81 or CD9 and CD151. Fig. S5. Overlap between CD9‐GFP and the nanobody labelling of CD9‐GFP. Fig. S6. Difference between diffraction limited and super‐resolution STED microscopy. [file FEB4-15-1678-s001.docx]

CD9-association with PIP_2_ areas is regulated by a CD9 salt bridge

Yahya Homsi^1^, Sara C. Konopka^1^, and Thorsten Lang^1,*^

^1^University of Bonn, Faculty of Mathematics and Natural Sciences, Membrane Biochemistry, Life & Medical Sciences (LIMES) Institute, Carl-Troll-Straße 31, 53115 Bonn, Germany

*Correspondence should be addressed to Thorsten Lang (thorsten.lang@uni-bonn.de)


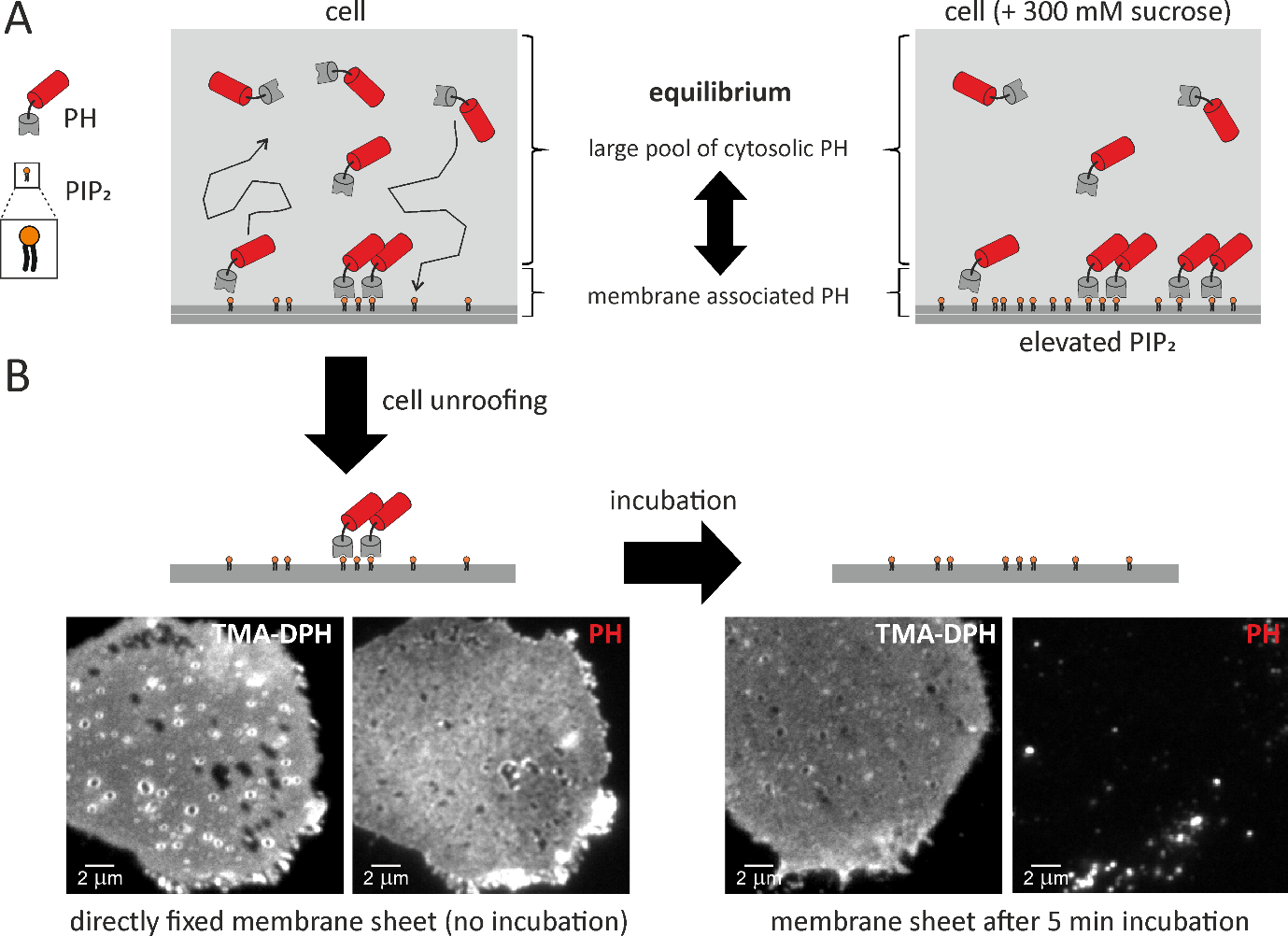


**Supplementary Figure 1.** *Illustration of the reversible binding of PH to PIP_2_.*

(A) Left panel, as the PH domain binds reversibly to the head group of PI(4,5)_2_, an equilibrium forms between membrane-associated and cytosolic PH. Right panel, after elevation of the PIP_2_-level more PH binding sites are available, and the equilibrium shifts towards more membrane-associated PH. (B) After cell unroofing, cytosolic PH does no longer bind, while membrane-associated PH dissociates (see micrographs for illustration). It takes a few minutes until PH has dissociated from the membrane (compare directly fixed to 5 min incubated membrane sheet). Fluorescence micrographs are shown at different settings of brightness and contrast, using a grayscale lookup table.


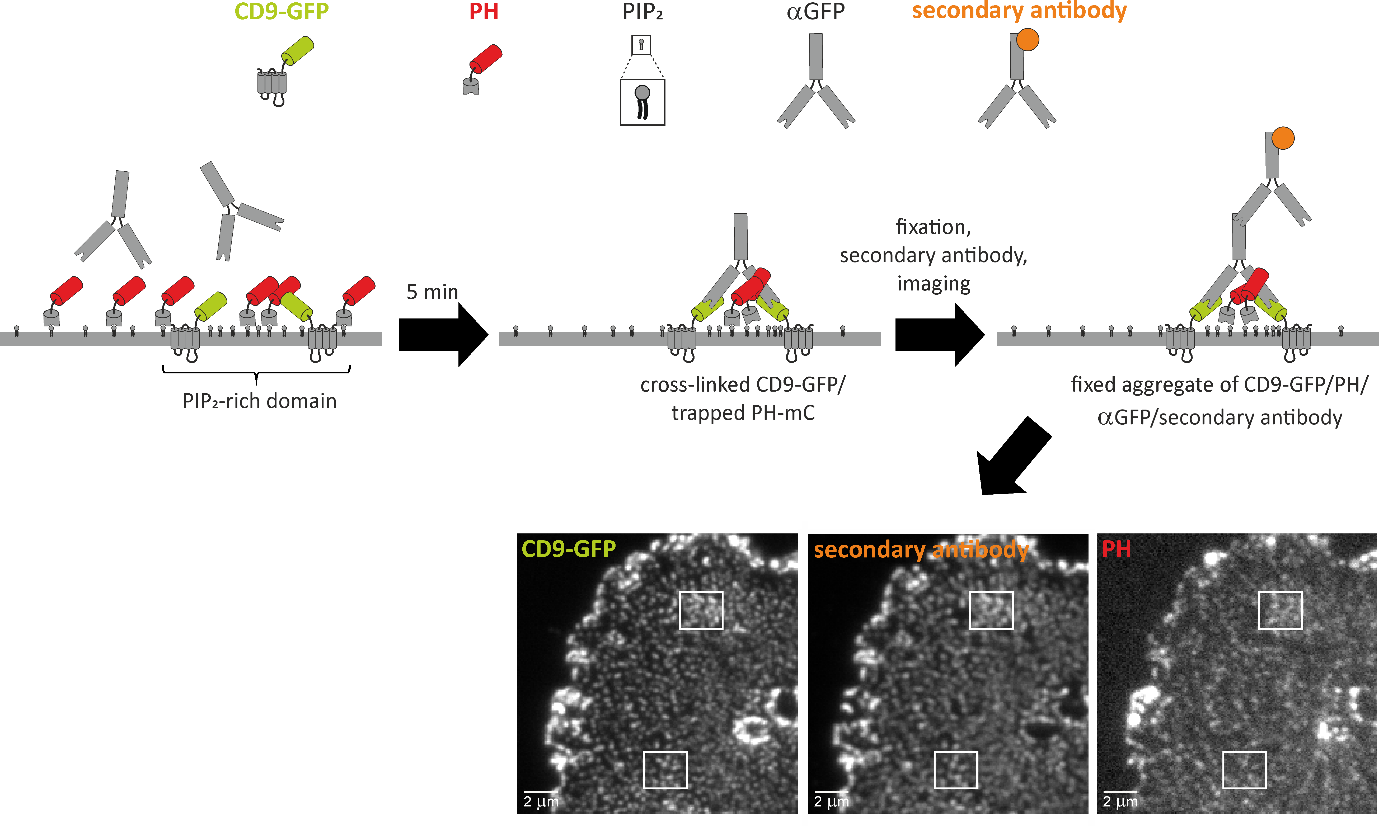


**Supplementary Figure 2.** *PH-trapping by GFP-antibody-induced CD9-GFP-aggregation.*

As illustrated in Supplementary Figure 1B, PH dissociates from the membrane within 5 min. Complete dissociation is prevented by the addition of a polyclonal GFP antibody (left). The antibody cross-links CD9-GFP molecules, and if PH is proximal, it becomes trapped within the aggregate (middle). After the 5 min incubation step, membranes are fixed and stained with a fluorescent labelled secondary antibody raised against the GFP-antibody (right). Overlap of the secondary antibody with CD9-GFP validates that the primary antibody has bound to GFP. Lower panels, for illustration, a micrograph of a membrane sheet incubated for 5 min with a GFP antibody followed by fixation and labeling with a secondary antibody is shown. From left to right, GFP-fluorescence (CD9-GFP), secondary antibody fluorescence, and PH-fluorescence. Please note that the retained PH locates at sites of aggregated proteins (CD9-GFP with bound GFP-antibody visualized by the secondary antibody). Boxes mark identical pixel locations and are a guidance for the eye. Fluorescence micrographs are shown at different settings of brightness and contrast, using a grayscale lookup table.


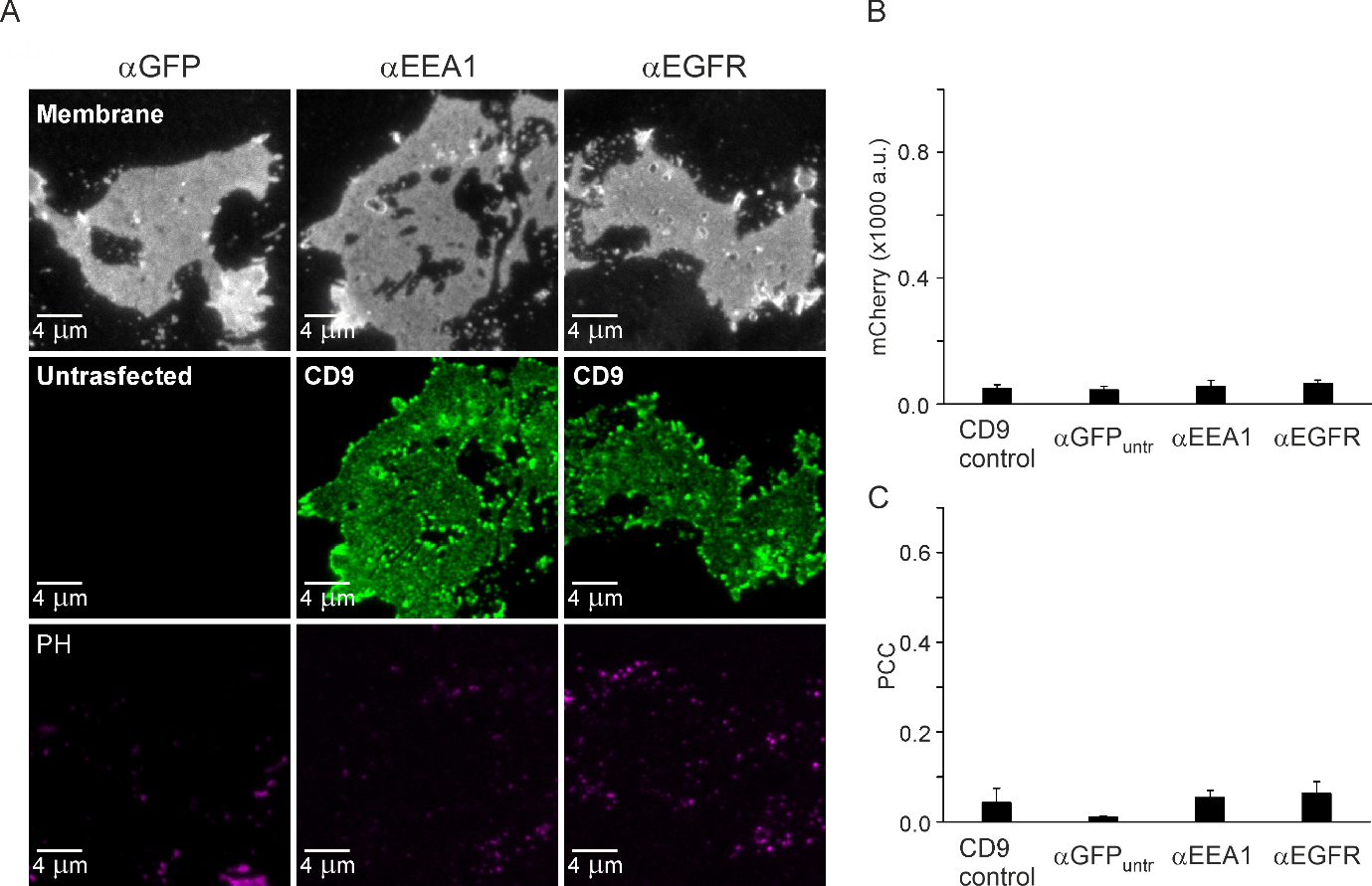


**Supplementary Figure 3.** *Antibody-patching control experiments.*

(A) Control experiments described in the figure legend of Figure 2. Untransfected (left) refers to the omittance of CD9-GFP plasmid in the transfection (only PH is expressed). Shown are membrane sheets incubated for 5 min with antibodies raised against GFP (αGFP), EEA1 (αEEA1) or EGFR (αEGFR). Top, membrane visualization by the dye TMA-DPH (displayed using a grayscale lookup table; images are shown at different settings of brightness and contrast). A green lookup table is used for CD9 (middle) and a magenta lookup table for PH (bottom). Images of CD9 and PH are shown at the same settings of brightness and contrast as the respective images of the same channels in Figure 2. (B) The membrane associated PH (mCherry-intensity) analyzed with squared ROIs. αGFP, αEEA1 and αEGFR are compared to a 5 min incubation without antibody (CD9 control, the same data as shown in Figure 2B). (C) PCCs between PH and CD9 analyzed with squared ROIs. αGFP, αEEA1 and αEGFR are compared to a 5 min incubation without antibody (CD9 control, the same data as shown in Figure 2C). Values are given as means ± SD (n = 3 biological replicates; each replicate and condition includes 12 - 21 analyzed membrane sheets).


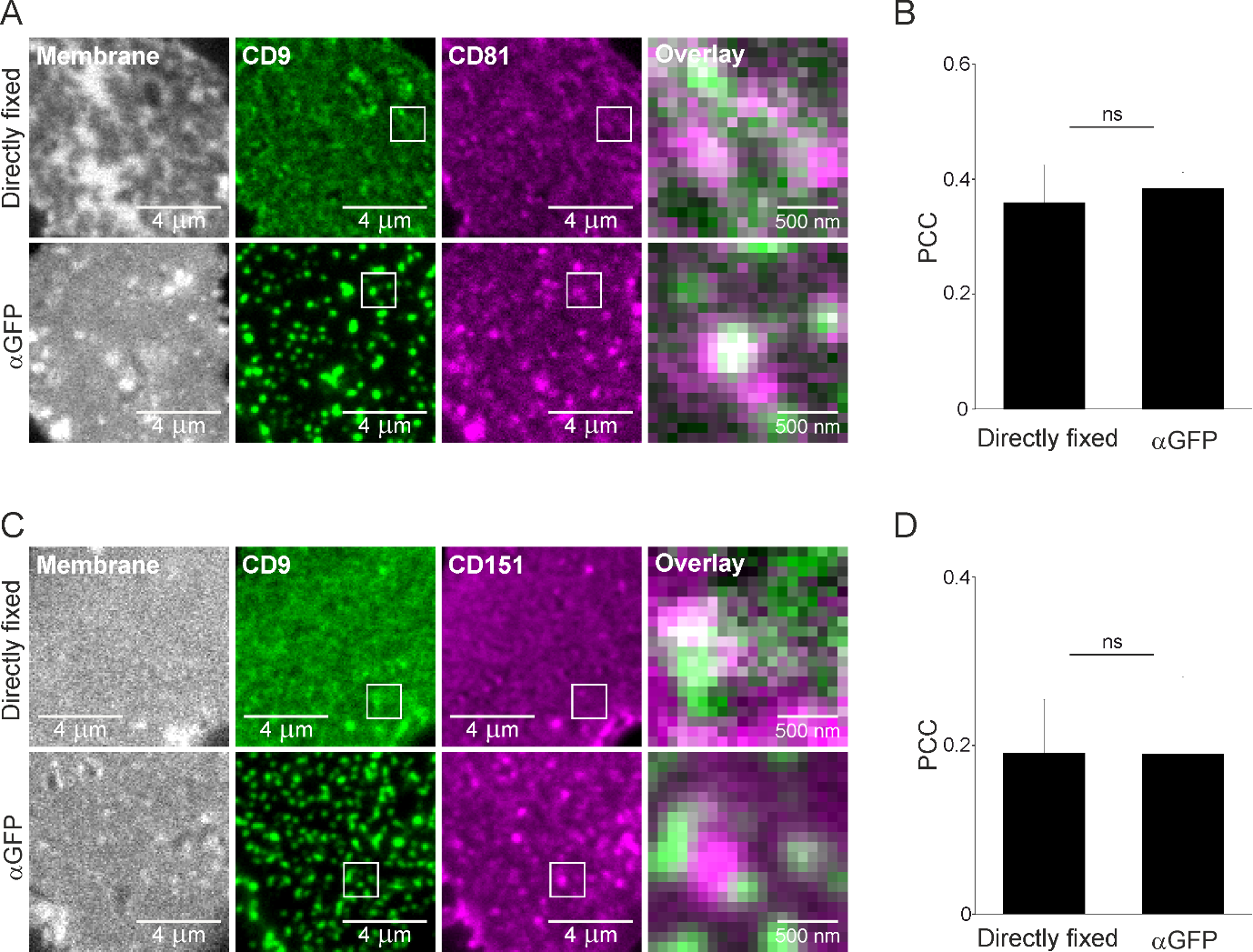


**Supplementary Figure 4.** *Antibody-induced CD9 patching does not affect the PCC between CD9 and CD81 or CD9 and CD151.*

HaCaT cells were double transfected with (A and B) CD9-GFP and CD81-RFP or (C and D) CD9-GFP and CD151-RFP. The next day, membrane sheets were generated and directly paraformaldehyde fixed (Directly fixed) or after a 5 min incubation at RT with an anti-GFP-antibody (αGFP). (A and C) Fluorescence micrographs are shown at different settings of brightness and contrast, using grayscale, green and magenta lookup tables. The average PCC between (B) CD9 and CD81 or (D) CD9 and CD151 were analyzed with squared ROIs. Values are given as means ± SD (n = 3 biological replicates; each replicate and condition includes 10 - 24 analyzed membrane sheets). Statistical test: unpaired two-tailed Student's t-test.


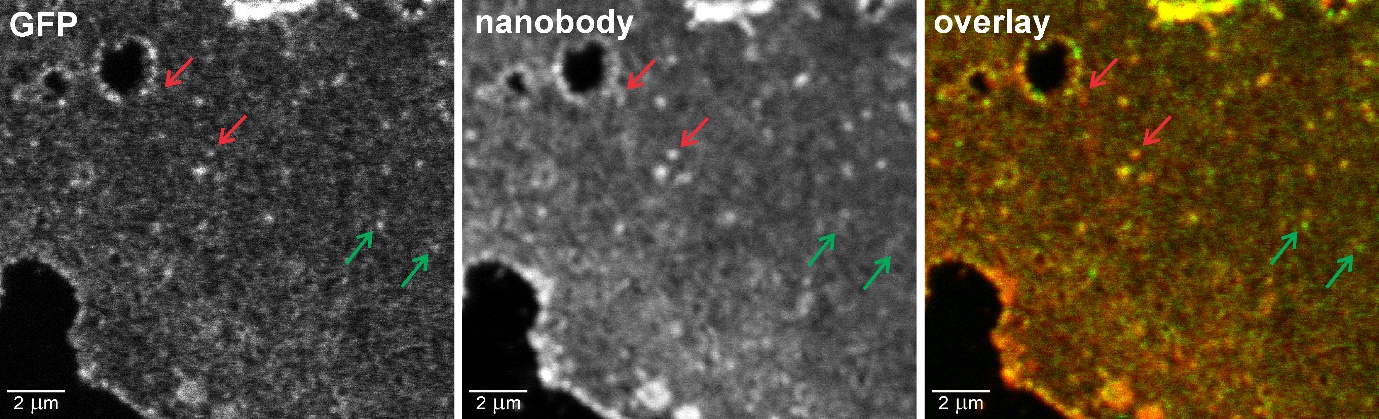


**Supplementary Figure 5.** *Overlap between CD9-GFP and the nanobody labeling of CD9-GFP.*

Shown are confocal micrographs that are routinely recorded prior to the STED micrographs. Left. the GFP-channel. Middle, the long-red channel that records the GFP-nanobody labeling. Right, overlay. The middle image has been corrected for bleedthrough from the red channel (detecting the nanobody-labelled CD9-RFP) into the long-red channel (see materials and methods). Fluorescence micrographs are shown at different settings of brightness and contrast, using a grayscale lookup table, and green and red lookup tables for the overlay. Arrows mark sites with uneven nanobody labelling. Green arrows: strong spots in the GFP channel are not noticed in the nanobody channel. Red arrows: rather strong nanobody labeling in comparison to the GFP signal. The PCC between the two channels was measured in squared ROIs, yielding a value of 0.37 ± 0.02. Values are given as means ± SD (n = 3 biological replicates; each replicate includes 10 - 16 analyzed membrane sheets).


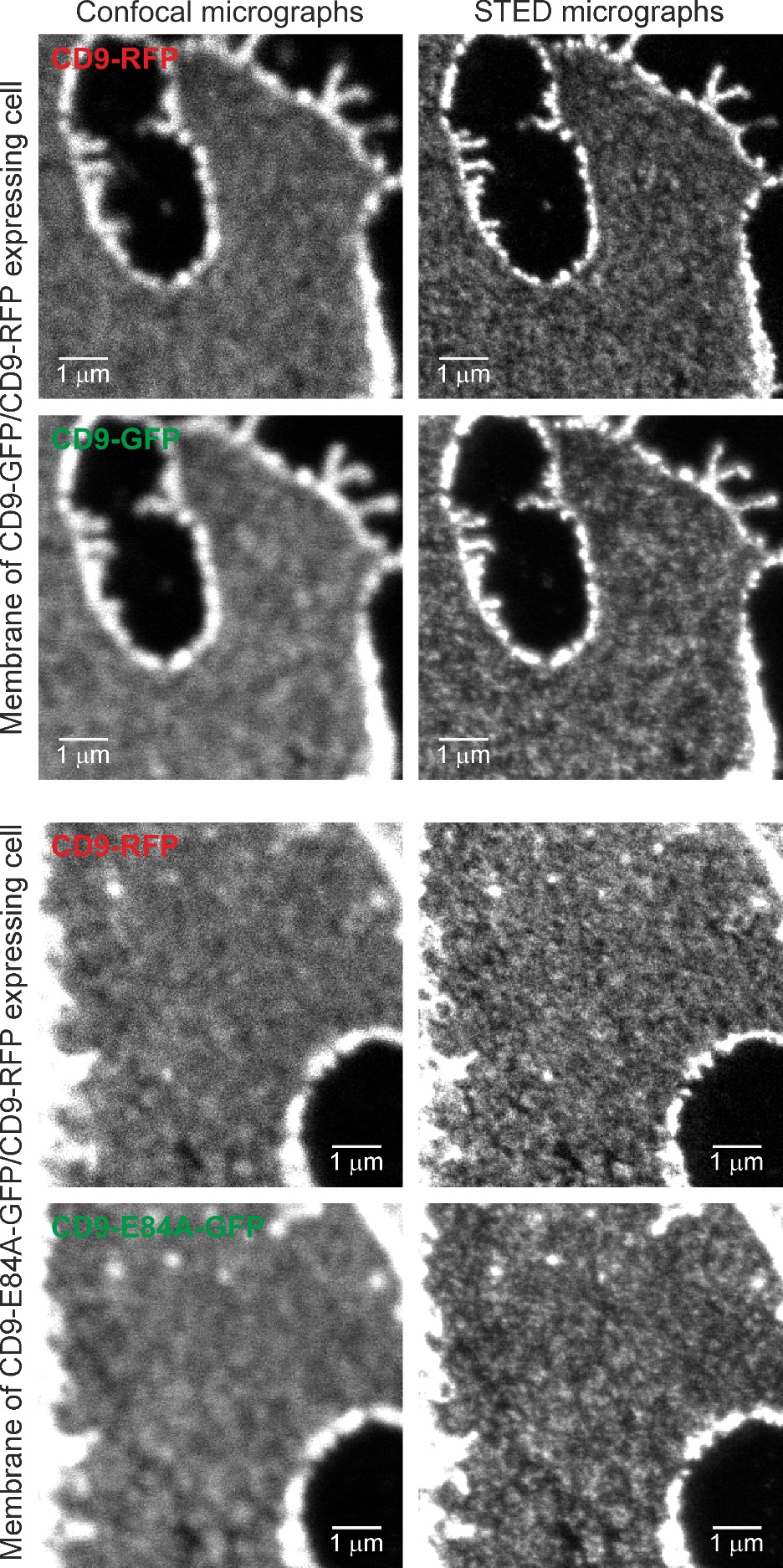


**Supplementary Figure 6.** *Difference between diffraction limited and superresolution STED microscopy.*

Left, confocal micrographs of the STED micrographs (right) shown in Figure 10. Images are shown at different settings of brightness and contrast.
